# Computational Investigation of a Weakly Coordinating Fluorinated Cosolvent as an SEI Contributor in Mixed-Solvent NaPF_6_ Electrolytes on Carbon Electrodes

**DOI:** 10.1021/acsomega.6c03386

**Published:** 2026-06-30

**Authors:** Paulo C. F. G. Neto, Fabio C. Romeu, Leonardo J. A. Siqueira, Luis G. Dias

**Affiliations:** † Chemistry Department, FFCLRP, University of São Paulo, 14040-901 São Paulo, Brazil; ‡ Hybrid Materials Laboratory, Chemistry Department, Institute of Environmental Science, Chemical, and Pharmaceutical, Federal University of São Paulo, 09913-030 São Paulo, Brazil; § Institute of Physics, University of Brasília, 70910-900 Brasilia, Brazil

## Abstract

In NaPF_6_-based triethyl phosphate–trifluoromethylbenzene–dimethyl carbonate electrolytes, trifluoromethylbenzene acts as a bulk solvent modifier that enhances salt solubility and facilitates sodium-ion transport. Using a combination of theoretical approaches that include classical molecular dynamics, reactive molecular dynamics in a tight-binding nanoreactor framework, and density functional theory with transition-state calculations, we investigated the role of trifluoromethylbenzene at the electrode–electrolyte interface. Despite its limited contribution to Na^+^ solvation, our results suggest that trifluoromethylbenzene can still promote the formation of a fluorine solid–electrolyte interphase through facilitated reductive decomposition at the electrode surface, leading to the release of fluoride ions.

## Graphical abstract

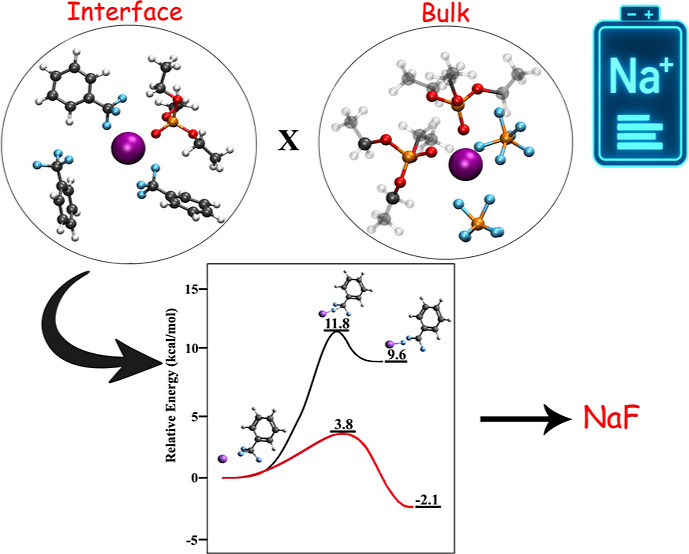

## 1 Introduction

Sodium-ion batteries have emerged as one of the most promising alternatives to lithium-ion systems, particularly in light of the rapidly increasing global demand for electrochemical energy storage and the economic and geopolitical constraints associated with lithium resources.^1^ In this context, the development of new materials and electrolytic systems with long cycle life, wide and stable electrochemical windows, and the ability to form a robust solid electrolyte interphase (SEI) is of critical importance to ensure safe and durable operation.^2–5^

Unlike Li^+^, the larger ionic radius and distinct solvation characteristics of Na^+^ introduce additional challenges related to electrode compatibility and electrolyte stability.^6^ In particular, the composition and nanostructure of the resulting inorganic-rich SEI depend on the reductive decomposition pathways of the electrolyte solvents, salts, and additives under interfacial electrochemical conditions. In addition, fluorine-containing species can significantly modify the interfacial chemistry and promote the formation of mechanically robust and ionically conductive SEI layers, thereby suppressing parasitic reactions at carbon-based electrodes. These findings reinforce the importance of understanding the electrolyte decomposition mechanisms at charged interfaces.

Building on these fundamental challenges, modern electrolyte design strategies have focused on engineering the molecular structure of solvents and precisely tuning cation solvent interactions to simultaneously improve ionic transport and ion solubility in the bulk, as well as interfacial stability.^7–12^ Mixtures with different polarities, dielectric constants, and coordination abilities allow fine control over the solvation energy and the ionic coordination number, regulating not only ionic mobility but also the ease with which the cation desolvates as it approaches the active electrode surface.^7–12^

Recently, Ma et al.^13^ proposed a ternary solvent mixture of triethyl phosphate (TEP), dimethyl carbonate (DMC), and trifluoromethylbenzene (PhCF_3_) as a flame-retardant electrolyte for NaPF_6_ salt (Figure 1), offering high voltage stability and wide temperature operability.

**Figure 1:**
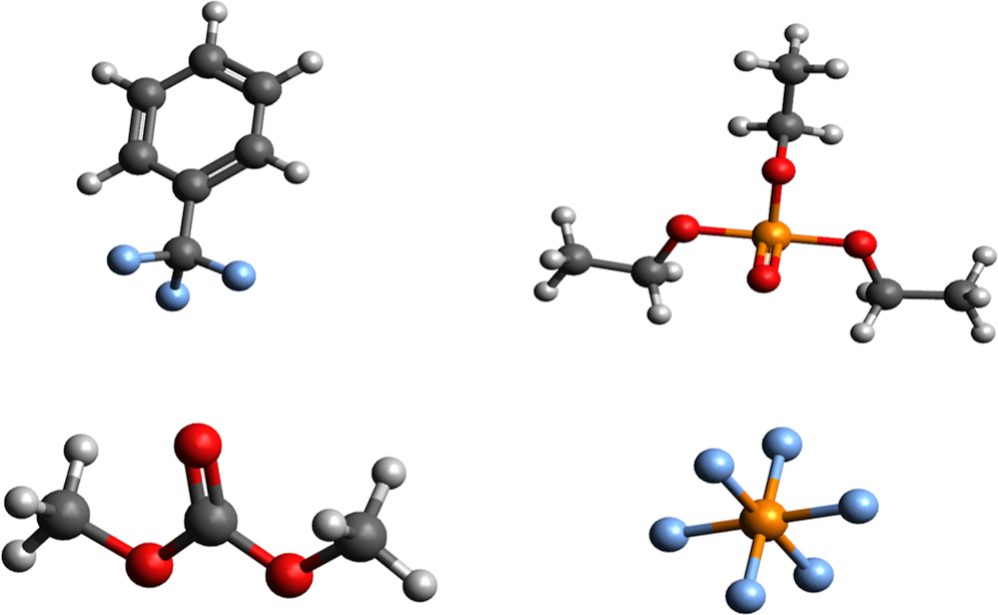
Ball-and-Stick representation of the species. From left to right, top row: trifluoromethylbenzene (PhCF_3_) and triethyl phosphate (TEP); bottom row: dimethyl carbonate (DMC) and hexafluorophosphate anion (PF_6_^–^). White, gray, cyan, red, and orange spheres represent hydrogen, carbon, fluorine, oxygen, and phosphorus atoms, respectively.

In the optimal molar ratio of 5 TEP: 3.5 PhCF_3_: 1 DMC, the electrolyte illustrates how competitive solvent–solvent and solvent–salt interactions can be tuned to enhance electrochemical performance. Notably, the authors demonstrated through molecular simulations that PhCF_3_ stabilizes TEP–Na^+^ aggregates without directly participating in ion coordination, instead acting as a promoter that facilitates solvent–ion coordination.^14^ The synergy among these components enhanced the ionic transport, stabilized SEI formation, and suppressed both structural phase transitions and transition-metal dissolution in the layered oxide cathode, ultimately improving long-term cycling stability.

We revisited the work of Ma et al.^13^ using a computational protocol that combines classical and reactive tight-binding molecular dynamics simulations with density functional theory calculations along reaction pathways, with particular emphasis on the organization of species at the electrode–electrolyte interface. The work of Ma et al.^13^ focused on the bulk solvation structure, ion transport, and electrochemical performance of the TEP/PhCF3/DMC electrolyte system, whereas the present work specifically investigates the interfacial organization and reductive decomposition pathways at charged carbon electrodes.

Thus, we discuss how the charged interface affects the solvation shell and local environment of Na^+^ ions, where initial reduction mechanism is expected between Na^+^ and PhCF_3_, and the associated activation energy. We propose that PhCF_3_ acts as a source of F^–^ ions at the interface, which subsequently contribute to the formation of the inorganic SEI.

In this context, the present results suggest that bulk coordination analysis alone may not be sufficient to predict interfacial stability or the SEI-forming propensity. More broadly, other weakly coordinating fluorinated cosolvents, diluents, or electrolyte additives may exhibit similar behavior, becoming selectively activated at the interface despite their limited role in bulk ion solvation. Such effects may be particularly important in localized high-concentration electrolytes and multicomponent solvent mixtures, where competitive intermolecular interactions and interfacial segregation can strongly influence reductive decomposition pathways and the final SEI composition.

## 2 Computational Methodology

To investigate how the charged interface influences the electrolyte’s spatial structure and its degradation pathways, we performed molecular dynamics simulations complemented by tight-binding and DFT calculations. The following sections describe the force fields, simulation protocols, and electronic structure methods employed in this work.

### 2.1 Force Field

We employed a modified version of the CHARMM36 General Force Field.^15^ The original parameters, when applied to the pure solvents, produced noticeable deviations from the experimental viscosities. To minimize these discrepancies, we empirically rescaled the Lennard–Jones parameters through a trial-and-error procedure, comparing the theoretical predictions of viscosity and density with the corresponding experimental data. We did not aim for a precise fit between experimental results and theoretical predictions; therefore, a semiquantitative agreement is expected, which is sufficient for the purposes of the present work. Details regarding the force-field recalibration and validation are provided in the Supporting Information. The complete set of recalibrated parameters is reported in Tables S1–S17, while the comparison between theoretical and experimental density and viscosity values is presented in Tables S19 and S24, respectively. Additional details about the viscosity calculations and fitting procedures are shown in Figures S2–S4.

### 2.2 Molecular Dynamics Simulations

The system consisted of a six-layer negatively charged graphene-like electrode (−0.0125|*e*| per carbon atom in the outermost sheets), generated using the Graphene Sheet Builder plugin in VMD,^16^ in contact with a solvent mixture in 5 TEP: 3.5 PhCF_3_: 1 DMC molar ratio, containing NaPF_6_ at a concentration of 1.0 mol L^–1^. The value of −0.0125|*e*| per carbon atom is within the range commonly employed in classical molecular dynamics studies of electrified carbon/electrolyte interfaces and should correspond to a potential slightly above 3.0 V.^17,18^ The boxes of 100 × 100 × 200 Å^3^ were constructed adopting a tolerance of 2.0 Å in PACKMOL.^19^ The system comprises graphene sheets placed at the center of the simulation box and kept fixed during the packing and simulation procedure, while the electrolyte components were distributed on both sides of the slab, excluding a central region in the vicinity of the surface. A similar symmetric single-electrode setup was recently adopted by us to investigate the sodium-ion insertion into a graphite-like carbon electrode model under constant-charge conditions.^17^ It should be noted that other setups can be found in the literature, such as two-electrode configurations under constant-potential^20–22^ or constant-electric-field^23^ conditions. The electrolyte consisted of 2000 TEP molecules, 1400 PhCF_3_ molecules, and 400 DMC molecules, together with 1096 PF_6_^–^ anions and 1096 Na^+^ cations. Additionally, 123 Na^+^ counterions were included to compensate the surface charge of the graphene sheet. All molecular species were randomly placed within the region defined by *z* = −100 to 100 Å, excluding the slab region between *z* = −9 and 9 Å. A snapshot of the system in its initial configuration is presented in Figure 2. A cubic bulk simulation box with dimensions of 100 × 100 × 100 Å^3^ was also simulated for comparative analysis with the interface results.

**Figure 2:**
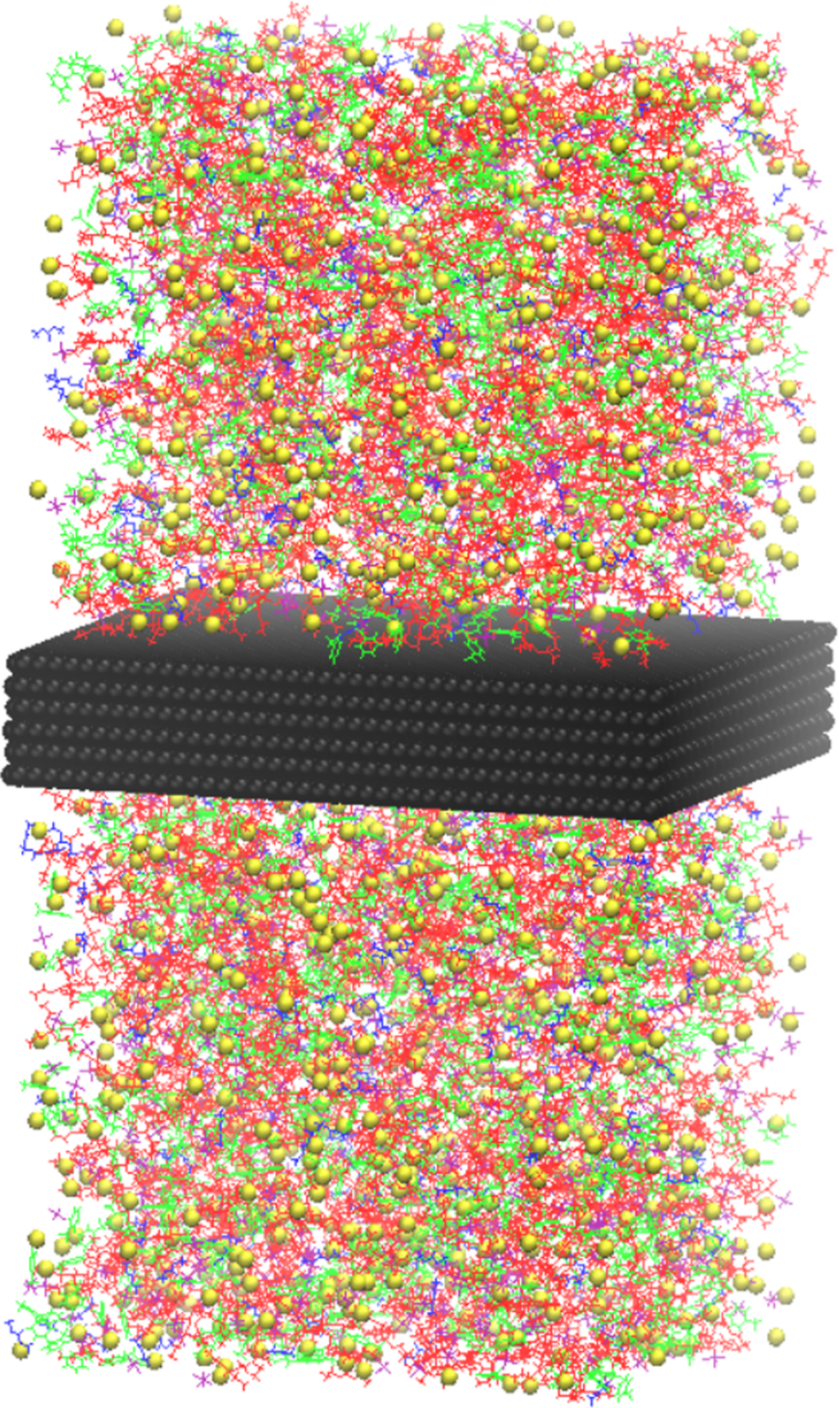
Initial molecular distribution in the simulation box. TEP, PhCF_3_, DMC, Na^+^, and PF_6_^–^ are shown in red, green, blue, yellow, and magenta, respectively. Graphene sheets are located at the box center, with carbon atoms in black color.

All simulations were performed with NAMD v2.14.^24^ Equilibration was first carried out in the *NVT* ensemble for 2 ns, followed by 4 ns in the NPzT ensemble. Subsequently, a 50 ns production run was performed in the NPzT ensemble. Lennard–Jones and real-space electrostatic interactions were truncated at 12 Å, with the Lennard–Jones potential smoothly switched between 10 Å and 12 Å. Long-range electrostatics were treated using the particle mesh Ewald (PME) method^25^ with a grid spacing of 1.0 Å and a sixth-order spline interpolation. The neighbor list was constructed with a pairlist distance of 16 Å and updated every 10 fs. Time integration was performed using a multiple time stepping scheme based on the r-RESPA algorithm,^26^ with a base integration time step of 2 fs. Short-range nonbonded interactions were evaluated every 4 fs, while long-range electrostatic interactions treated with PME were computed every 8 fs. Covalent bonds involving hydrogen atoms were constrained using the SHAKE algorithm.^27^ Temperature was maintained at 298 K using a Langevin thermostat^28^ with a damping constant of 1.0 ps^–1^, and pressure was controlled at 1 bar using a Langevin piston barostat^29^ with a damping constant of 10 ps^–1^.

The number density profiles of the different species along the *z* coordinate, radial distribution functions, and the coordination numbers around Na^+^ ions in both the bulk and interfacial regions were computed using in-house codes and LOOS.^30^

### 2.3 Density Functional Tight-Binding in Nanoreactor Framework

Possible reaction pathways were investigated using molecular dynamics simulations at the GFN2-xTB^31^ level for 100 ps, employing a spherical nanoreactor.^32,33^ This reactor was designed to confine the system and facilitate dissociation processes during the simulations. In our study, the confining potential was set using the logfermi wall with the instruction sphere: auto, all, which allows xTB to determine the radius automatically from the molecular geometry. The automatic radius is computed from the largest interatomic distance in the structure plus an internal offset, ensuring that the system remains within a physically meaningful volume. The spherical logfermi potential is centered at the origin (0,0,0). Thus, the confinement acts relative to this fixed point unless the coordinates are shifted typically via the $cma command to place the molecular center of mass at the origin. This setup ensures proper placement of the system within the confining sphere while avoiding artificial interactions with the wall.

The molecular dynamics were propagated with 1 fs integration steps using the velocity Verlet algorithm^34^ and Berendsen thermostat^35^ with a coupling constant of 0.1 ps. In this stage, one electron was introduced to probe potential reductive bond-cleavage events at open-shell simulations. Python scripts were built to identify species present before and after bond cleavage based on Wiberg bond order^36^ calculations. Mulliken population analysis^37^ was also performed on the fragments to identify neutral or charged species. All tight-binding calculations were performed with xTB 6.7.1.^38^

### 2.4 Density Functional Theory with Transition-State Search

The bond cleavage process was examined using the Nudge elastic band (NEB)^39^ at the UB3LYP-D4 ^40–42^ level with def2-SVP basis set^43^ to obtain an initial approximation of the reaction path passing through the transition state. The transition-state structure was subsequently refined through the OPT-TS method^44^ at the same level of theory.

Intrinsic reaction coordinate (IRC)^45^ calculations were performed to verify the connectivity of the located transition state with the corresponding reactant and product minima. The IRC calculations were carried out at the UB3LYP-D4/def2-SVP level starting from the fully optimized transition-state geometry, previously characterized by the presence of a single imaginary vibrational frequency. The reaction path was followed in both the forward and backward directions by using the mass-weighted IRC formalism to confirm that the transition state smoothly connects the intended reactant and product wells on the potential energy surface, thereby validating the proposed reaction mechanism.

Single-point calculations included solvent effects via the conductor-like polarizable continuum model (C-PCM)^46^ with the Gaussian charge scheme and scaled vdW-type cavity,^47^ using an effective dielectric constant of approximately 10. The value is slightly higher than those of TEP (ϵ_*r*_ = 8.55) and PhCF_3_ (ϵ_*r*_ = 9.18) at *T* = 293 K,^48^ thereby providing an upper-bound estimate for the mixed solvent environment. Nonelectrostatic contributions to the solvation free energy were neglected in the C-PCM calculations.

All DFT calculations were performed with ORCA 5.0.4.^49^

## 3 Results and Discussion

### 3.1 Structural Organization Preceding Interfacial Reactivity

In the uncharged state of the electrode, PhCF_3_ molecules are found to adsorb at the surface (green line in the top panel of Figure 3). The corresponding number density profile displays two well-defined peaks, at *z* = 11.4 Å and *z* = 13.3 Å, which can be assigned to two distinct molecular orientations: a flat-lying configuration, in which the phenyl ring remains approximately parallel to the surface, and a slightly tilted configuration. This assignment is supported by the peak position of the carbon atom located at the para position relative to the CF_3_ group. Specifically, this peak is slightly shifted along the *z*-direction relative to the first fluorine peak. The preference for the flat-lying configuration can be rationalized by the maximization of Lennard–Jones interactions with the carbon surface. Sodium ions (orange line) and DMC (blue line) molecules are practically absent at the uncharged electrode surface. Only a minor presence is observed for TEP (red line) molecules, whereas PF_6_^–^ (magenta line) anions are found at the interface.

**Figure 3:**
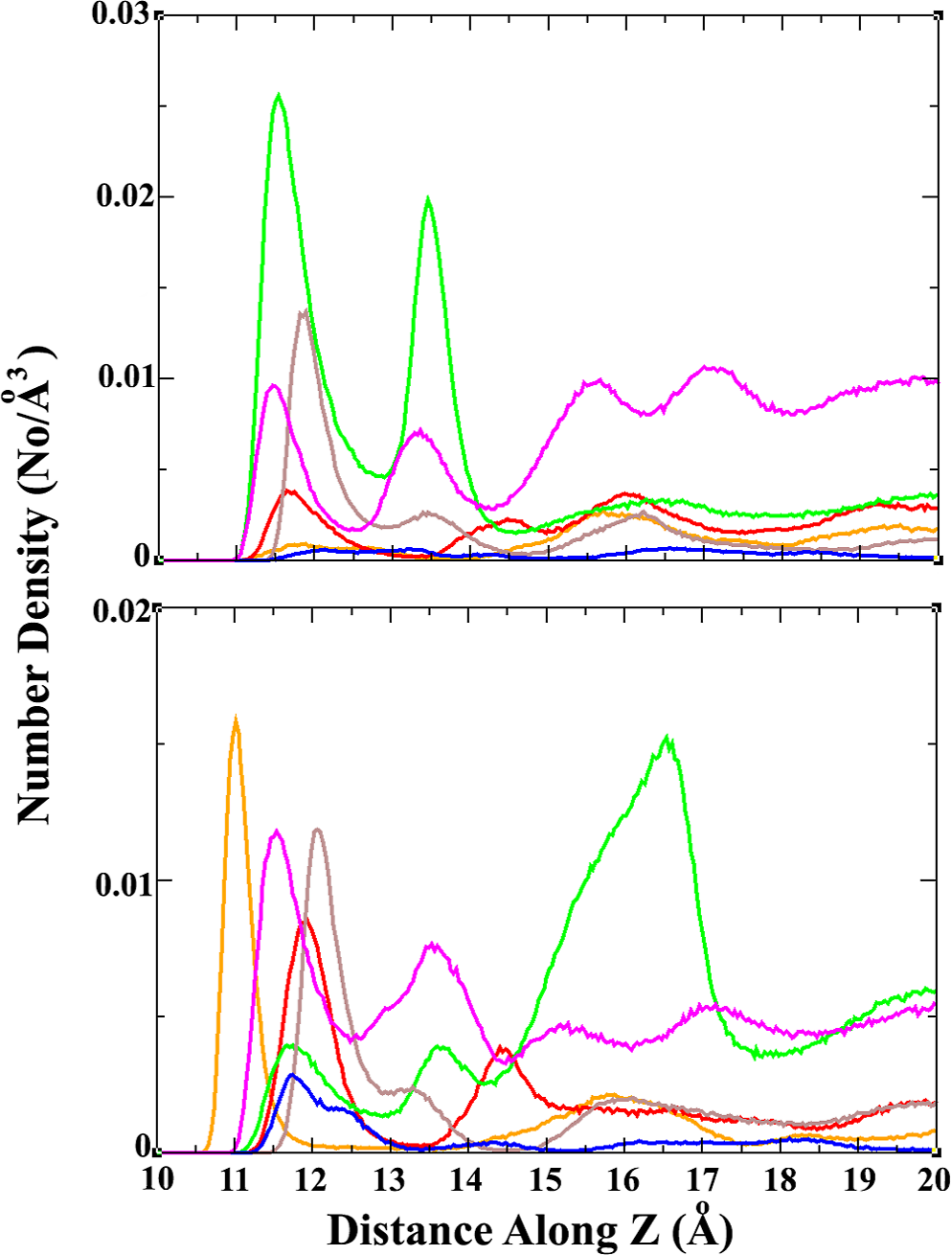
Number density profiles of fluorine atoms (green line) and carbon atom located at the para position relative to the CF_3_ group (brown line), Na^+^ ions (orange line), oxygen atoms of the carbonate group in DMC (blue line), oxygen atoms of the phosphate group in TEP (red line), and fluorine atoms from PF_6_^–^ (magenta line), near charged (bottom) and neutral (top) carbon electrodes in NaPF_6_ 1.0 mol L^–1^.

Upon charging the surface, Na^+^ cations are attracted to the negatively charged electrode via electrostatic interactions, leading to the formation of a compact Na^+^ adlayer at the electrode surface. TEP and DMC molecules are also found at the interface due to the strong interaction with the Na^+^ ions. In contrast, PF_6_^–^ anions are attracted to the interface in an attempt to screen the excess of positive charge. PhCF_3_ molecules are displaced to a second interfacial layer adjacent to the Na^+^ adlayer (bottom panel of Figure 3), with the excess population located beyond 15.0 Å. The presence of PhCF_3_ molecules at the interface is mainly governed by Lennard–Jones interactions with the electrode atoms.

The interface alters the solvation shell of Na^+^ ions, as depicted by the RDFs shown in Figure 4. In the bulk, the RDF between Na^+^ and PhCF_3_ molecules indicates that PhCF_3_ is absent from the first solvation shell. Instead, the carbon atoms located at the para position with respect to the CF_3_ group are found closer to the Na^+^ ions than the fluorine atoms. Although PhCF_3_ molecules adopt a tilted configuration, at the interface, the RDF between fluorine atoms and Na^+^ presents an important correlation ≈2.5 Å, which indicates their direct interaction.

**Figure 4:**
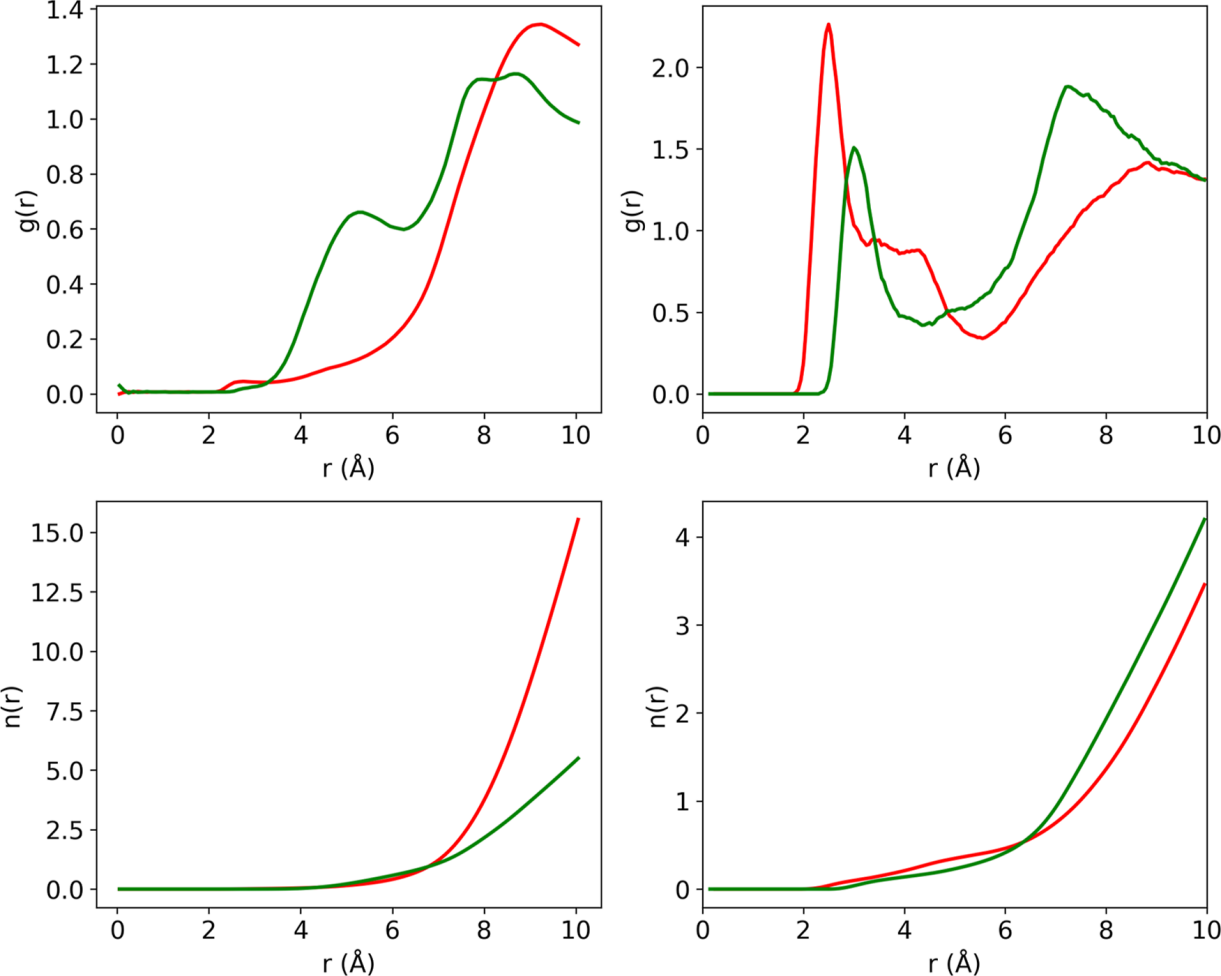
Radial distribution functions, *g*(*r*) (upper panels), and integrated coordination numbers, *n*(*r*) (lower panels), for the fluorine atoms of the CF_3_ group (red line) and the carbon atom located at the para position with respect to the CF_3_ group (green line) in the bulk phase (left panels) and at the interface (right panels).

### 3.2 Nanoreactor Insights into Solvent Decomposition Mechanisms

Although the average coordination number of PhCF_3_ around Na^+^ ions is not high, the spatial proximity between the cation and the CF_3_ groups of PhCF_3_ raises the hypothesis that these species may engage in a reactive event during electron transfer at the charged interface. To investigate this possibility, we employed the nanoreactor approach based on MD simulations at the GFN2-xTB level, which enables accelerated sampling of rare reactive events under thermally driven fluctuations.

Within this reactive framework, C–F bond activation was observed during the time evolution of the Na-PhCF_3_ system upon addition of one excess electron at 400 K. Wiberg bond orders and Mulliken atomic charges were monitored throughout the simulation for all bonds. The time evolution of the C–F Wiberg bond order and the Mulliken atomic charge on the fluorine atom are shown in Figure 5.

**Figure 5:**
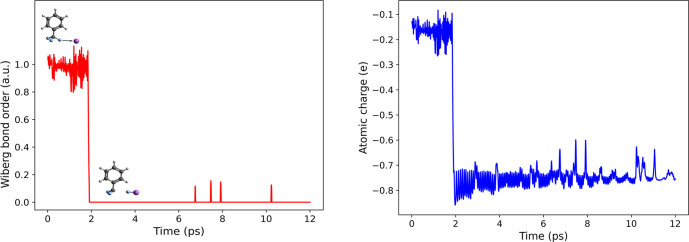
C–F Wiberg bond order (left panel) and fluorine Mulliken atomic charge (right panel) as a function of simulation time. The top panel inset emphasizes the bond-breaking event by highlighting the configurations before and after the reaction.

Clearly, we can observe a change in the bond order from approximately 1 to 0 within about 2 ps of MD simulation, indicating cleavage of the single C–F bond. In addition, the atomic charge changes from approximately −0.15|*e*| to −0.8|*e*|, indicating that the fluorine species leaving the PhCF_3_ molecule is the F^–^ anion.

In addition, we also investigated redox events involving TEP and DMC species, confirming decomposition pathways consistent with previous studies reported in the literature^50,51^ and supporting the reliability of our protocol. It is important to clarify that only the C–F bond cleavage pathway was observed in our nanoreactor simulations for the PhCF_3_ + Na system. Specifically, we carried out 10 independent reactive trajectories to investigate whether alternative reductive decomposition mechanisms could emerge under the simulation conditions. However, in all cases, the same heterolytic C–F bond cleavage event was consistently observed, leading to fluoride anion formation, while no competing reaction pathways were identified. We have clarified this point in the Supporting Information together with the corresponding bond-order and fragment-charge analyses (Figures S10 and S11).

### 3.3 Mechanistic Insights into Electrolyte Bond-Cleavage Processes

Using the Na–PhCF_3_ pair as the reference for the reduction reaction, the relative activation energy for C–F bond scission is approximately 12 kcal/mol (Figure 6). The vibrational normal mode associated with the transition state (Figure 7) corresponds to C–F bond cleavage, with the fluorine atom displaced toward the sodium ion. Exceptionally, in the presence of a moderate polar medium, both transition state and products are substantially stabilized (Figure 6). Therefore, the substantial reduction in the barrier for the PhCF_3_ + Na^+^ redox reaction indicates that C–F bond activation becomes kinetically accessible under mild interfacial conditions. Notice that we have used a relatively modest basis set and level of theory for the intrinsic reaction coordinate calculations, while the continuum solvent description does not explicitly capture local solvent organization effects that may influence the reaction energetics at the electrode/electrolyte interface. However, the primary objective of the present DFT calculations is not to provide quantitatively converged activation energies but rather to validate the mechanistic plausibility of the C–F bond cleavage pathway identified in the nanoreactor simulations. In this context, the selected level of theory offers a reasonable compromise between computational cost and reliability for exploring reaction pathways and transition states. Importantly, the calculations consistently predict a kinetically accessible C–F bond cleavage process and a thermodynamically favorable fluoride formation.

**Figure 6:**
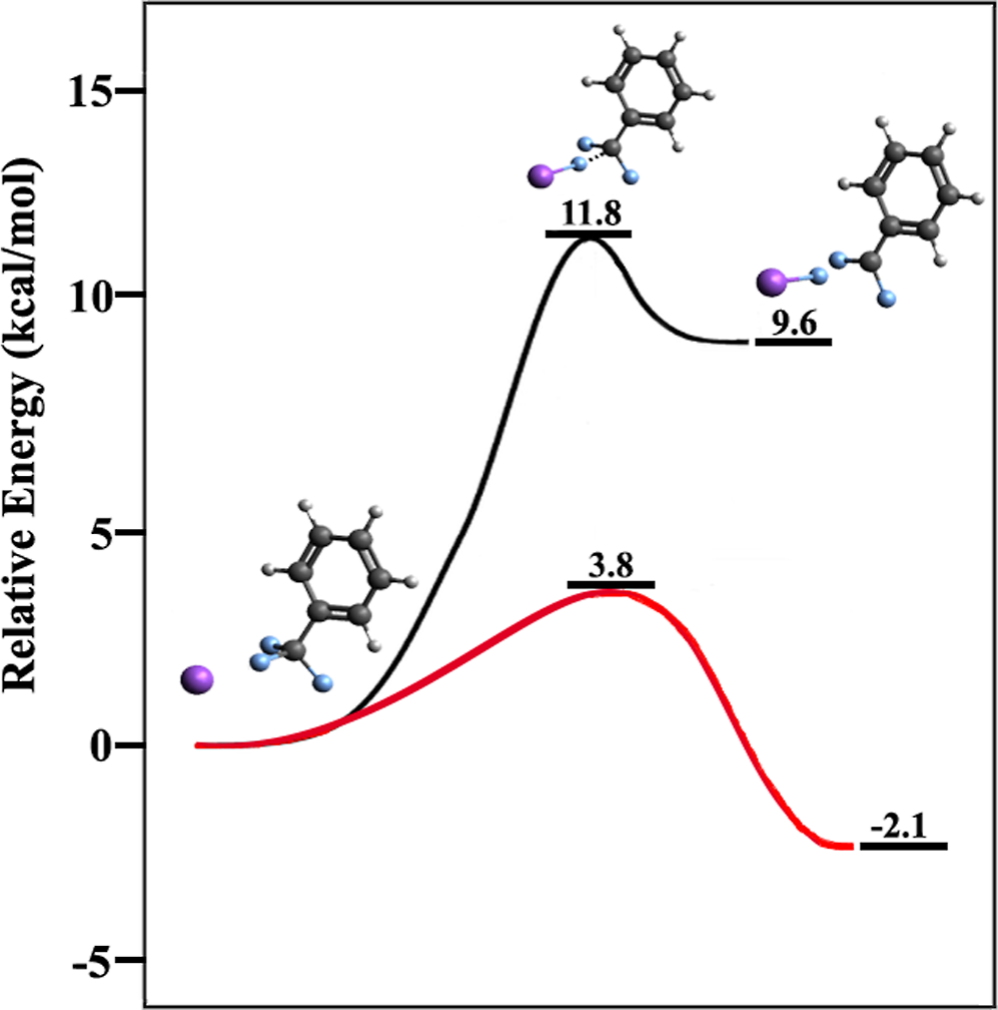
Intrinsic reaction coordinate toward the reagents and products of the Na^+^ + PhCF_3_ reduction reaction calculated at the UB3LYP-D4/def2-SVP level. Energies are reported relative to the reactants. Atom colors follow Figure 1, with Na^+^ shown as a magenta sphere. The red curve corresponds to the calculation including solvation effect, whereas the black curve refers to the gas-phase calculation.

**Figure 7:**
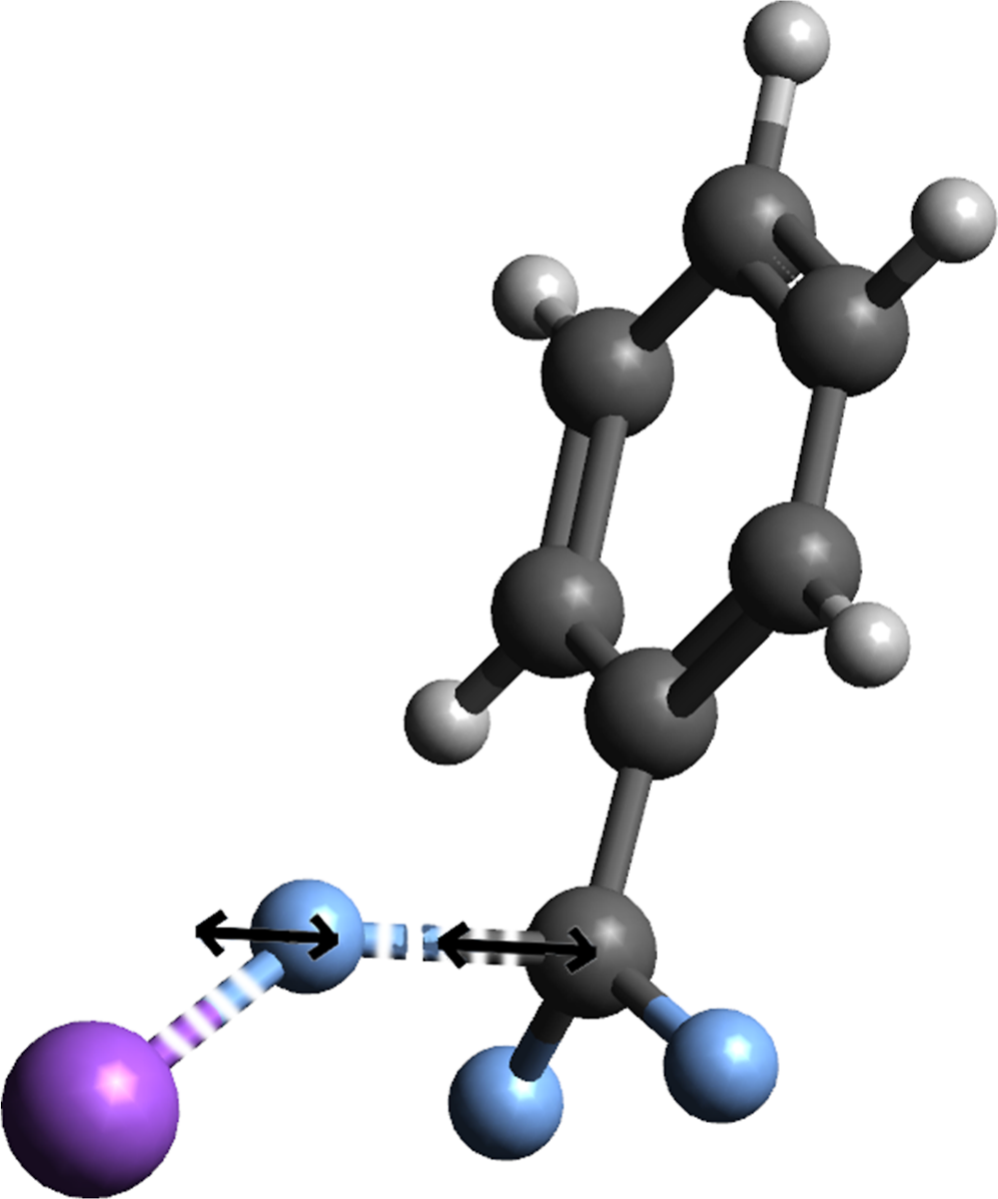
Geometry of the transition state for C–F bond scission. Black vectors represent the normal vibrational mode associated with the imaginary frequency of 322.95 cm^–1^. Calculations were performed at the UB3LYP-D4/def2-SVP level.

In addition, we evaluated the solvent reaction field effect for the TEP and DMC decomposition reactions (see the Supporting Information). In these cases, the continuum solvent effects were minimal.

We also investigated a reaction pathway for fluoride anion generation arising from the PF_6_^–^ dissociation process under continuum solvation conditions

1 $${{PF}_{6}}^{-}\to F^{-}+{PF}_{5}$$

The process could take place in an organic solvent; however, the calculated energy difference between products and reactants is 58.3 kcal/mol at the B3LYP-D4­(C-PCM)/def2-QZVPP//B3LYP-D4­(C-PCM)/def2-SVP level of theory. This value is consistent with previous results reported by Fattebert and Alzate-Vargas for the decomposition of PF_6_^–^ in the presence of Li^+^, with both species solvated in ethylene carbonate.^52^ In principle, such a high barrier effectively excludes this decomposition pathway under mild conditions, raising intriguing questions regarding the mechanism of F^–^ generation from PF_6_^–^ in this organic medium that warrants future investigation.

## 4 Conclusions and Perspectives

The results of this study demonstrate that, although PhCF_3_ is a Na^+^-ion weakly coordinating solvent, it can play an important role at the charged electrode interface, where it must undergo decomposition and release fluoride anions. These fluoride species, together with sodium ions, act in the formation of a stable inorganic-rich SEI layer.

We would like to clarify that the mechanism proposed in the present work should be interpreted within the specific context of carbonaceous electrodes and that direct extrapolation to other anode materials, such as sodium metal, should be made with caution. Under such conditions, additional decomposition pathways may become accessible, including more extensive solvent reduction that is not necessarily mediated by the same interfacial organization effects discussed here.

Furthermore, this work presents a computational protocol capable of elucidating electrolyte reduction mechanisms in complex, multicomponent systems, highlighting the role of charged interfaces in modulating reactivity. Understanding how interfacial structure and local ion–molecule arrangements influence reductive stability provides valuable insights for the rational design of multifunctional electrolytes. Ongoing work aims to further refine the protocol and improve the force field, enabling in silico development of electrolyte formulations with controlled bulk and interfacial properties.

## Supplementary Material



## Notes

Additional information about the computational approach, force field evaluation for density and viscosity, nanoreactor results at tight-binding levels, RDFs in the bulk and interface, and NEB and IRC calculations at the DFT level (PDF)
