# Supplementary material for: Computational Investigation of a Weakly Coordinating Fluorinated Cosolvent as an SEI Contributor in Mixed-Solvent NaPF6 Electrolytes on Carbon Electrodes
Source: ACS Omega. 2026 Jun 30;11(27):40460–7. doi: 10.1021/acsomega.6c03386 (PMC13382819; doi:10.1021/acsomega.6c03386)
Supplement: Supplementary file 1 [file ao6c03386_si_001.pdf]

Supporting Information

Computational Investigation of a Weakly Coordinating Fluorinated Co-Solvent as an SEI Contributor in Mixed-Solvent $NaPF_6$ Electrolytes on Carbon Electrodes

Paulo C. F. G. Neto[1], Fabio C. Romeu[1], Leonardo J. A. Siqueira[2,3], Luis G. Dias[1,*]

[1]Department of Chemistry, FFCLRP, University of São Paulo, Ribeirão Preto, SP, Brazil

[2]Hybrid Materials Laboratory, Department of Chemistry, Institute of Environmental, Chemical and Pharmaceutical Sciences, Federal University of São Paulo, Diadema, SP, Brazil

[3]Institute of Physics, University of Brasília, Brasília, DF, Brazil

**Corresponding author:* lgdias@ffclrp.usp.br

Contents

1 Additional Information about the Computational Approach **3**

2 Additional Results **10**

2.1 Force Field Evaluation and Recalibration 10

2.1.1 Density 10

2.1.2 Viscosity 11

2.2 Radial Distribution Functions 16

2.3 Exploring Reaction Pathways in the Nanoreactor 18

2.3.1 TEP + Na reaction 18

2.3.2 $PhCF_3$ + Na reaction 20

2.3.3 DMC + Na reaction 22

2.4 Transition-state Calculations 24

2.4.1 Nudged Elastic Band 24

2.4.2 Intrinsic Reaction Coordinate 26

3 References **29**

1 Additional Information about the Computational Approach

The CHARMM force field [1] splits the interactions into bonded and nonbonded terms. The bonded terms are given by a bond stretching harmonic potential, an angle bending harmonic potential, a dihedral torsion potential, an improper dihedral potential, and the Urey–Bradley 1,3-interaction. The non-bonded terms are given by the Lennard–Jones (LJ) 6–12 potential, and the Coulomb potential between atomic monopoles. The Equation 1 shows the expression of the total potential:

$$\begin{aligned} U_{\text{total}} = &\sum_{\text{bonds}} k_b(b-b_0)^2 + \sum_{\text{angles}} k_\theta(\theta-\theta_0)^2 + \sum_{\text{dihedrals}} k_\varphi\left[1+\cos(n\varphi-\delta)\right] \\ &+ \sum_{\text{improper}} k_\phi(\phi-\phi_0)^2 + \sum_{\text{Urey-Bradley}} k_{UB}(r_{1,3}-r_{1,3,0})^2 \\ &+ \sum_{\text{nonbonded}} \left[\frac{q_i q_j}{4\pi\varepsilon_0 r_{ij}} + \epsilon_{ij}\left(\left(\frac{R_{\min,ij}}{r_{ij}}\right)^{12} - 2\left(\frac{R_{\min,ij}}{r_{ij}}\right)^{6}\right)\right] \end{aligned} \tag{1}$$

The intramolecular portion of the potential energy function includes terms for the bonds, valence angles, torsion or dihedral angles, improper dihedral angles, and a Urey–Bradley 1,3-term, where b_0, θ_0, ψ_0, and $r_{1,3,0}$ are the bond, angle, improper, and Urey–Bradley equilibrium terms, respectively, n and δ are the dihedral multiplicity and phase, and the k's are the respective force constants.

The intermolecular terms include q_i and q_j are the partial atomic charges of atoms i and j, respectively, ϵ_{ij} is the well depth, $R_{\min,ij}$ is the radius in the LJ 6–12 term used to treat the vdW interactions, and r_{ij} is the distance between atoms i and j. To implicitly account for polarization and charge-transfer effects not explicitly included in the CHARMM force field, the ionic net charges were uniformly scaled by a factor of 0.8.[2, 3]

Tables 1–17 list the force field parameters. For the vdW interactions, the values of

σ are reported instead of $R_{\min}$. The two are related by $R_{\min} = \sqrt[6]{2}\sigma$. Figure 1 shows the three-dimensional representation of the molecules, including their atom types.

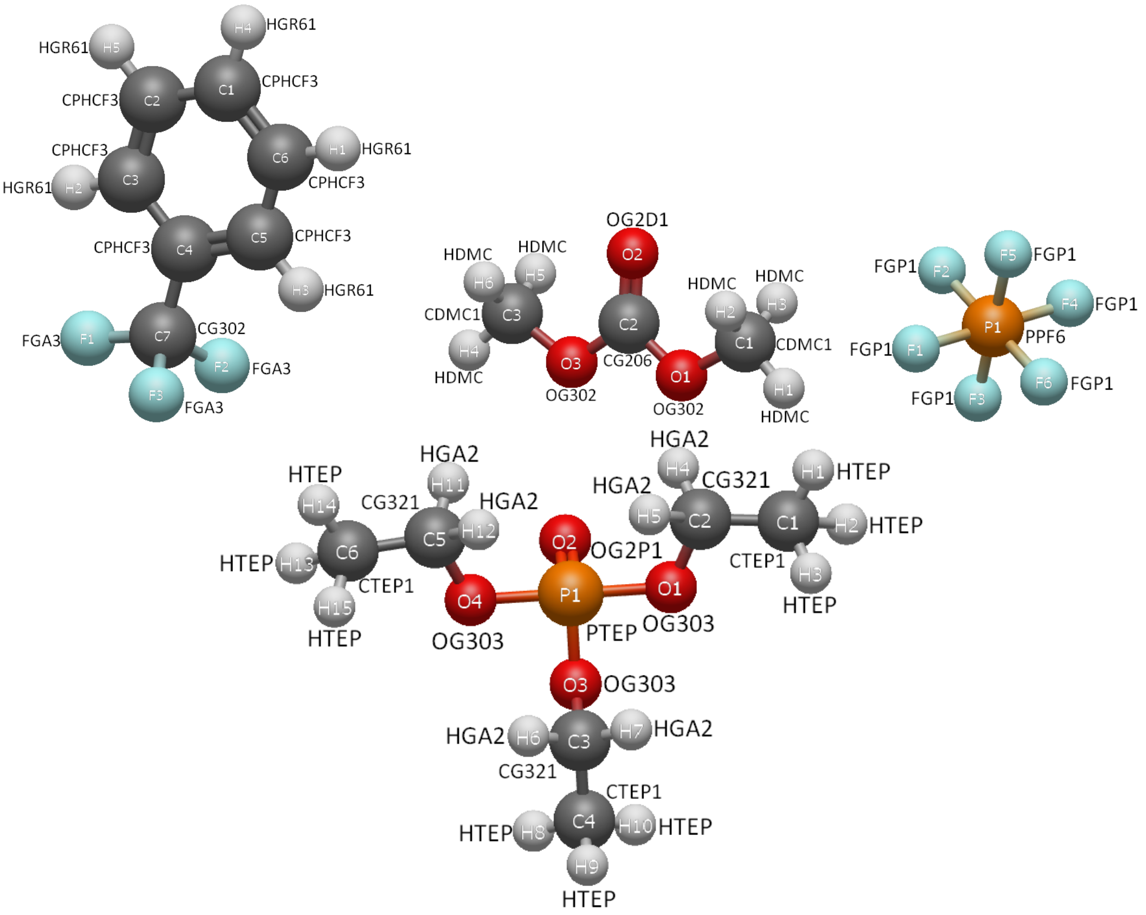

Figure S1: Ball-and-stick model of the solvent system with triethylphosphate (TEP), trifluoromethylbenzene ($PhCF_3$), dimethyl carbonate (DMC), and the hexafluorophosphate anion (PF_6^-). Atom colors: H (white), C (gray), O (red), F (light blue), P (orange). Atom types are also depicted.

Table S1: Lennard–Jones parameters and atomic charges for $PhCF_3$.

Atom	Atom Type	σ (Å)	ϵ (kcal·mol^{-1})	q
C1	CPHCF3	2.09202	-0.0630	-0.170
C2	CPHCF3	2.09202	-0.0630	-0.140
C3	CPHCF3	2.09202	-0.0630	-0.140
C4	CPHCF3	2.09202	-0.0630	-0.140
C5	CPHCF3	2.09202	-0.0630	-0.170
C6	CPHCF3	2.09202	-0.0630	-0.010
C7	CG302	2.41500	-0.0180	0.630
F1	FGA3	1.68000	-0.0873	-0.220
F2	FGA3	1.68000	-0.0873	-0.220
F3	FGA3	1.68000	-0.0873	-0.220
H1	HGR61	1.42611	-0.0270	0.150
H2	HGR61	1.42611	-0.0270	0.160
H3	HGR61	1.42611	-0.0270	0.180
H4	HGR61	1.42611	-0.0270	0.160
H5	HGR61	1.42611	-0.0270	0.150

Table S2: Lennard–Jones parameters and atomic charges for DMC.

Atom	Atom Type	σ (Å)	ϵ (kcal·mol^{-1})	q
C1	CDMC1	2.0705	-0.0663	-0.035
C2	CG2O6	2.0200	-0.0595	1.180
C3	CDMC1	2.0705	-0.0663	-0.035
O1	OG302	1.6665	-0.0850	-0.500
O2	OG2D1	1.7170	-0.1020	-0.680
O3	OG302	1.6665	-0.0850	-0.500
H1	HDMC	1.3534	-0.0204	0.095
H2	HDMC	1.3534	-0.0204	0.095
H3	HDMC	1.3534	-0.0204	0.095
H4	HDMC	1.3534	-0.0204	0.095
H5	HDMC	1.3534	-0.0204	0.095
H6	HDMC	1.3534	-0.0204	0.095

Table S3: Lennard–Jones parameters and atomic charges for $NaPF_6$.

Atom	Atom Type	σ (Å)	ϵ (kcal·mol^{-1})	q
Na	SOD	1.41075	-0.0469	0.800
P	PPF6	2.15000	-0.5850	0.880
F1	FGP1	1.60000	-0.0970	-0.280
F2	FGP1	1.60000	-0.0970	-0.280
F3	FGP1	1.60000	-0.0970	-0.280
F4	FGP1	1.60000	-0.0970	-0.280
F5	FGP1	1.60000	-0.0970	-0.280
F6	FGP1	1.60000	-0.0970	-0.280

Table S4: Lennard–Jones parameters and atomic charges for TEP.

Atom	Atom Type	σ (Å)	ϵ (kcal·mol^{-1})	q
P	PTEP	2.1715	-0.4095	1.260
O1	OG303	1.6665	-0.0700	-0.520
O2	OG2P1	1.7170	-0.0840	-0.660
O3	OG303	1.6665	-0.0700	-0.520
O4	OG303	1.6665	-0.0700	-0.520
C1	CTEP1	2.0705	-0.0546	-0.400
C2	CG321	2.0301	-0.0392	0.380
C3	CG321	2.0301	-0.0392	0.380
C4	CTEP1	2.0705	-0.0546	-0.400
C5	CG321	2.0301	-0.0392	0.380
C6	CTEP1	2.0705	-0.0546	-0.400
H1	HTEP	1.3534	-0.0168	0.100
H2	HTEP	1.3534	-0.0168	0.100
H3	HTEP	1.3534	-0.0168	0.100
H4	HGA2	1.3534	-0.0245	0.020
H5	HGA2	1.3534	-0.0245	0.020
H6	HGA2	1.3534	-0.0245	0.020
H7	HGA2	1.3534	-0.0245	0.020
H8	HTEP	1.3534	-0.0168	0.100
H9	HTEP	1.3534	-0.0168	0.100
H10	HTEP	1.3534	-0.0168	0.100
H11	HGA2	1.3534	-0.0245	0.020
H12	HGA2	1.3534	-0.0245	0.020
H13	HTEP	1.3534	-0.0168	0.100
H14	HTEP	1.3534	-0.0168	0.100
H15	HTEP	1.3534	-0.0168	0.100

Table S5: Bond lenght parameters for $PhCF_3$ ·

Bond	r_0 (Å)	k_b (kcal.mol^{-1}.Å^{-2})
CPHCF3–CPHCF3	1.3750	305.0
CPHCF3–CG302	1.4500	198.0
CPHCF3–HGR61	1.0800	340.0
CG302–FGA3	1.3400	265.0

Table S6: Bond lenght parameters for DMC.

Bond	r_0 (Å)	k_b (kcal.mol^{-1}.Å^{-2})
CG2O6–OG2D1	1.2300	650.0
CG2O6–OG302	1.3500	350.0
CDMC1–OG302	1.4300	340.0
CDMC1–HDMC	1.1110	322.0

Table S7: Bond lenght parameters for PF_6^- ·

Bond	r_0 (Å)	k_b (kcal.mol^{-1}.Å^{-2})
PPF6–FGP1	1.5800	237.0

Table S8: Bond lenght parameters for TEP.

Bond	r_0 (Å)	k_b (kcal.mol^{-1}.Å^{-2})
CG321–CTEP1	1.5280	222.5
CG321–OG303	1.4400	320.0
CG321–HGA2	1.1110	309.0
CTEP1–HTEP	1.1110	322.0
OG2P1–PTEP	1.4800	580.0
OG303–PTEP	1.6100	230.0

Table S9: Angle and Urey–Bradley parameters for $PhCF_3$ ·

Angle	θ_0 (°)	k_θ (kcal.mol^{-1}.rad^{-2})	$r_{1,3,0}$ (Å)	k_{UB} (kcal.mol^{-1}.Å^{-2})
CPHCF–CPHCF3–CPHCF3	120.0	40.0	2.41620	35.00
CPHCF3–CPHCF3–CG302	120.0	45.8	-	-
CPHCF3–CPHCF3–HGR61	120.0	30.0	2.15250	22.00
CPHCF3–CG302–FGA3	115.0	50.0	2.35700	30.00
FGA3–CG302–FGA3	107.0	118.0	2.15500	30.00

Table S10: Angle and Urey–Bradley parameters for DMC.

Angle	θ_0 (°)	k_θ (kcal.mol^{-1}.rad^{-2})	$r_{1,3,0}$ (Å)	k_{UB} (kcal.mol^{-1}.Å^{-2})
OG2D1–CG2O6–OG302	123.5	70.0	-	-
OG302–CG2O6–OG302	105.0	85.0	-	-
OG302–CDMC1–HDMC	109.5	60.0	-	-
HDMC–CDMC1–HDMC	108.4	35.5	1.80200	5.40
CG2O6–OG302–CDMC1	111.0	40.0	-	-

Table S11: Angle and Urey–Bradley parameters for PF_6^- .

Angle	θ_0 (°)	k_θ (kcal.mol^{-1}.rad^{-2})	$r_{1,3,0}$ (Å)	k_{UB} (kcal.mol^{-1}.Å^{-2})
FGP1–PPF6–FGP1	108.0	48.1	-	-

Table S12: Angle and Urey–Bradley parameters for TEP.

Angle	θ_0 (°)	k_θ (kcal.mol^{-1}.rad^{-2})	$r_{1,3,0}$ (Å)	k_{UB} (kcal.mol^{-1}.Å^{-2})
CTEP1–CG321–OG303	108.4	70.0	-	-
CTEP1–CG321–HGA2	110.1	34.6	2.17900	22.53
OG303–CG321–HGA2	109.5	60.0	-	-
HGA2–CG321–HGA2	109.0	35.5	1.80200	5.40
CG321–CTEP1–HTEP	110.1	34.6	2.17900	22.53
HTEP–CTEP1–HTEP	108.4	35.5	1.80200	5.40
CG321–OG303–PTEP	120.0	20.0	2.33000	35.00
OG2P1–PTEP–OG303	111.6	98.9	-	-
OG303–PTEP–OG303	108.0	48.1	-	-

Table S13: Dihedral potential parameters for $PhCF_3$.

Dihedral	δ (°)	n	V_n (kcal.mol^{-1})
CPHCF3–CPHCF3–CPHCF3–CPHCF3	180.0	2	3.1000
CPHCF3–CPHCF3–CPHCF3–CG302	180.0	2	3.1000
CPHCF3–CPHCF3–CPHCF3–HGR61	180.0	2	4.2000
CG302–CPHCF3–CPHCF3–HGR61	180.0	2	2.4000
HGR61–CPHCF3–CPHCF3–HGR61	180.0	2	2.4000
CPHCF3–CPHCF3–CG302–FGA3	0.0	2	0.3000

Table S14: Dihedral potential parameters for DMC.

Dihedral	δ (°)	n	V_n (kcal.mol^{-1})
OG2D1–CG2O6–OG302–CDMC1	180.0	1	0.2500
OG2D1–CG2O6–OG302–CDMC1	180.0	2	1.8500
OG2D1–CG2O6–OG302–CDMC1	0.0	3	0.1200
OG302–CG2O6–OG302–CDMC1	180.0	1	0.5500
OG302–CG2O6–OG302–CDMC1	180.0	2	2.9500
HDMC–CDMC1–OG302–CG2O6	0.0	3	0.0000

Table S15: Dihedral potential parameters for TEP.

Dihedral	δ (°)	n	V_n (kcal.mol^{-1})
OG303–CG321–CTEP1–HTEP	0.0	3	0.1600
HGA2–CG321–CTEP1–HTEP	0.0	3	0.1600
CTEP1–CG321–OG303–PTEP	180.0	1	0.6000
CTEP1–CG321–OG303–PTEP	0.0	2	0.6500
CTEP1–CG321–OG303–PTEP	0.0	3	0.0500
HGA2–CG321–OG303–PTEP	0.0	3	0.0000
CG321–OG303–PTEP–OG2P1	0.0	3	0.1000
CG321–OG303–PTEP–OG303	0.0	3	0.5000

Table S16: Improper torsion parameters for $PhCF_3$ ·

Improper	ω_0 (°)	k_ω (kcal.mol^{-1}.rad^{-2})
CPHCF3–CPHCF3–CPHCF3–CPHCF3	0.0	20.0075
CPHCF3–CPHCF3–CPHCF3–CG302	180.0	20.0075
CPHCF3–CPHCF3–CPHCF3–HGR61	180.0	20.0075
HGR61–CPHCF3–CPHCF3–HGR61	0.0	20.0075

Table S17: Improper torsion parameters for DMC.

Improper	ω_0 (°)	k_ω (kcal.mol^{-1}.rad^{-2})
CG2O6–OG302–OG302–OG2D1	0.0	145.0000

2 Additional Results

2.1 Force Field Evaluation and Recalibration

The CHARMM General force field [1] shows deviations from experimental densities and viscosities for the pure compounds $PhCF_3$, TEP, and DMC[4, 5, 6, 7]. To improve agreement with experiment, the Lennard–Jones parameters were scaled by trial and error to reduce the observed discrepancies. We do not aim for a precise fit between experiments and theoretical predictions; therefore, a semi-quantitative agreement is expected. The total absolute relative error (TARE) for density and viscosity was adopted as the error metric and minimized during parameters optimization:

$$\text{TARE} = |\frac{\rho_{\text{calc}} - \rho_{\text{exp}}}{\rho_{\text{exp}}}| + |\frac{\eta_{\text{calc}} - \eta_{\text{exp}}}{\eta_{\text{exp}}}| \tag{2}$$

2.1.1 Density

To calculate the densities of pure solvent, different initial cubic boxes were generated using Packmol[8] as indicated in the Table S18.

Table S18: System size for density evaluation.

System	Molecules number	Lateral box size (Å)
TEP	442	50.21
$PhCF_3$	608	49.85
DMC	894	49.92

The densities were obtained from molecular dynamics (MD) simulations performed with NAMD v2.14 [9] in the NPT ensemble. Lennard–Jones and real-space electrostatic interactions were truncated at 12.0 Å. The Lennard–Jones potential was smoothly switched between 10.0 Å and the cutoff distance. Long-range electrostatics were treated using the particle–mesh Ewald (PME) method [10] with a grid spacing of 1.0 Å and a sixth-order spline interpolation. The neighbor list was constructed with a pairlist

distance of 16 Å and updated every 10 fs. A 1 fs integration timestep was employed, with the SHAKE algorithm used to constrain covalent bonds involving hydrogen atoms [11]. Temperature was maintained at 298 K using a Langevin thermostat [12] with a damping constant of 1.0 ps^{-1}, and pressure was controlled at 1 bar using a Langevin piston barostat [13] with a damping constant of 10 ps^{-1}. All systems were first energy-minimized for 3000 steps, followed by a 3 ns NPT equilibration run and a 2 ns NPT production run.

Table S19: Comparison of density values between the modified CHARMM, original CHARMM and experimental data at 298 K.

System	$\rho_{original}$ ($g.cm^{-3}$)	ρ_{new} ($g.cm^{-3}$)	ρ_{exp} ($g.cm^{-3}$)
$PhCF_3$	1.266	1.160 ± 0.001	1.180
DMC	1.075	1.013 ± 0.001	1.069
TEP	1.127	1.022 ± 0.001	1.068

2.1.2 Viscosity

To calculate the viscosities of pure solvent, different initial cubic boxes were generated using PACKMOL[8] as indicated in the Table S20.

Table S20: System size for viscosity evaluation.

System	Molecules number	Lateral box size (Å)
TEP	4420	109.34
$PhCF_3$	6080	108.32
DMC	8940	109.72

The shear viscosity of the pure substances was determined from MD simulations performed with NAMD v2.14 [9] in the NVE ensemble [14]. The cutoff scheme for short-range interactions and the treatment of long-range electrostatics followed the same parameters described in the density section. The neighbor list construction and time integration algorithm were also identical. Pressure was evaluated at the atomic virial level with the system center of mass removed. Each system was first energy-minimized

for 3000 steps, equilibrated in the NPT ensemble for 2 ns using a Langevin thermostat (damping constant of 1.0 ps^{-1}) and Langevin piston barostat (damping constant of 10.0 ps^{-1}), and finally simulated in the NVE ensemble for a 1 ns production run.

The shear viscosity was calculated from the integral over time of the pressure tensor autocorrelation function:

$$\eta = \frac{V}{k_B T} \int_0^\infty \langle P_{\alpha\beta}(0) P_{\alpha\beta}(t) \rangle \, dt \tag{3}$$

The procedure closely follows to the methodology proposed by Zhang, Otoni and Maginn[15]:

1. Generate a number of independent NVE trajectories at the target temperature.

2. Calculate the shear viscosity for each trajectory using Eq. 2.

3. Compute the average and standard deviation over the trajectories:

$$\sigma_\eta(t) = \sqrt{\frac{1}{N-1} \sum_{i=1}^{N} \left[\eta_i(t) - \langle \eta(t) \rangle\right]^2}. \tag{4}$$

4. Fit the averaged running integral with a sum of exponentials functions:

$$\sum_i A_i \left(1 - e^{-t/T_i}\right) \tag{5}$$

   weighted by the $\frac{1}{\sigma_\eta^2}$

5. The equilibrium shear viscosity is then obtained by:

$$\sum_i A_i \tag{6}$$

The results for independent NVE trajectories of DMC, $PhCF_3$ and TEP at 298 K are presented in the figures and tables below. The theoretical modified CHARMM

(η_{new}) and CHARMM original ($\eta_{original}$) values for shear viscosity against experimental data are shown in Table S24.

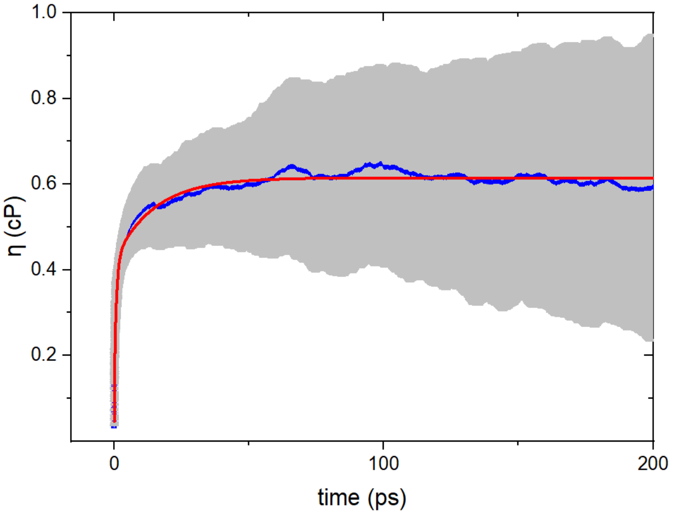

Figure S2: Time convergence of shear viscosity for DMC. Blue: mean curve of 20 independent trajectories; gray shadow: dispersion generated from 20 independent trajectories; red: fitted curve.

Table S21: Statistical parameters of the exponential fits to the mean viscosity curve for DMC.

	Number of exponential functions		
Parameters	1	2	3
R^2	0.57305	0.91194	0.91981
χ^2_ν	5.40918	1.11566	1.01598
A_1	0.473 ± 0.001	0.426 ± 0.000	0.286 ± 0.001
A_2	-	0.144 ± 0.000	0.137 ± 0.000
A_3	-	-	0.192 ± 0.001
t_1	0.251 ± 0.001	1.612 ± 0.006	0.796 ± 0.006
t_2	-	0.003 ± 0.000	0.002 ± 0.000
t_3	-	-	14.738 ± 0.242
η	0.473 ± 0.001	0.570 ± 0.001	0.615 ± 0.001

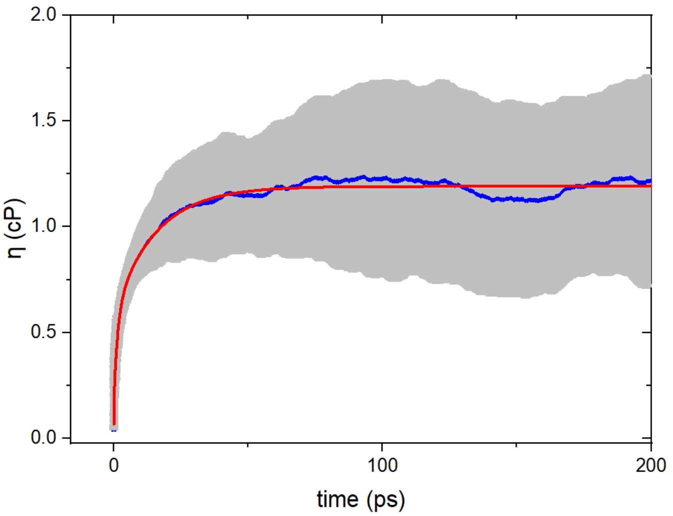

Figure S3: Time convergence of shear viscosity for $PhCF_3$. Blue: mean curve of 10 independent trajectories; gray shadow: dispersion generated from 10 independent trajectories; red: fitted curve.

Table S22: Statistical parameters of the exponential fits to the mean viscosity curve for $PhCF_3$.

	Number of exponential functions		
Parameters	1	2	3
R^2	0.62305	0.87804	0.88755
χ^2_ν	5.32278	1.72213	1.58789
A_1	0.924 ± 0.001	0.923 ± 0.001	0.602 ± 0.004
A_2	-	0.196 ± 0.000	0.180 ± 0.000
A_3	-	-	0.409 ± 0.004
t_1	1.047 ± 0.004	4.885 ± 0.017	15.384 ± 0.210
t_2	-	0.002 ± 0.000	0.002 ± 0.000
t_3	-	-	1.419 ± 0.019
η	0.924 ± 0.001	1.120 ± 0.001	1.191 ± 0.004

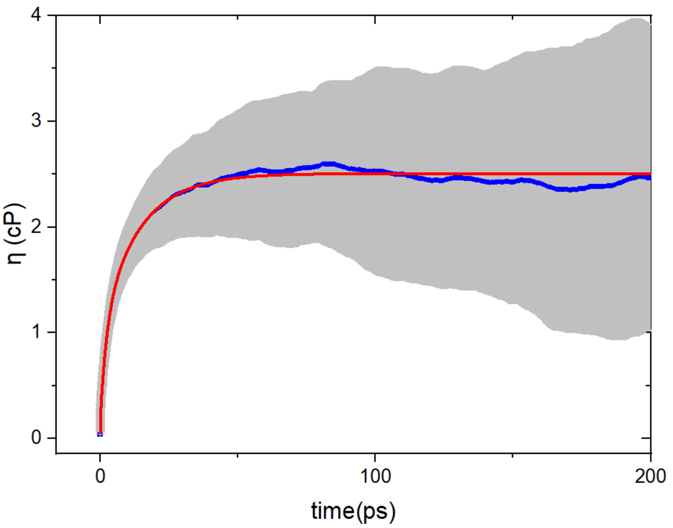

Figure S4: Time convergence of shear viscosity for TEP. Blue: mean curve of 10 independent trajectories; gray shadow: dispersion generated from 10 independent trajectories; red: fitted curve.

Table S23: Statistical parameters of the exponential fits to the mean viscosity curve for TEP.

	Number of exponential functions		
Parameters	1	2	3
R^2	0.68911	0.94584	0.95104
χ^2_ν	5.26709	0.91758	0.82951
A_1	2.037 ± 0.003	2.149 ± 0.002	1.453 ± 0.010
A_2	-	0.208 ± 0.000	0.197 ± 0.000
A_3	-	-	0.857 ± 0.011
t_1	2.383 ± 0.008	5.789 ± 0.010	13.971 ± 0.146
t_2	-	0.003 ± 0.001	0.003 ± 0.001
t_3	-	-	2.314 ± 0.026
η	2.037 ± 0.003	2.357 ± 0.002	2.507 ± 0.011

Table S24: Comparison of shear viscosity between the modified CHARMM, original CHARMM and experimental data at 298 K.

System	η_{original} (cP)	η_{new} (cP)	η_{exp} (cP)
TEP	37.946	2.507 ± 0.011	1.600
$PhCF_3$	1.596	1.191 ± 0.004	0.551
DMC	1.057	0.615 ± 0.001	0.589

2.2 Radial Distribution Functions

Radial distribution functions (RDFs) between Na^+ ions and the oxygen atom of the phosphate group in TEP, the oxygen atom of the carbonate group in DMC and the fluorine atoms of the $PF_6{}^-$ anion. In general, interfacial RDFs resemble bulk RDFs but display smaller coordination numbers around Na^+ ions.

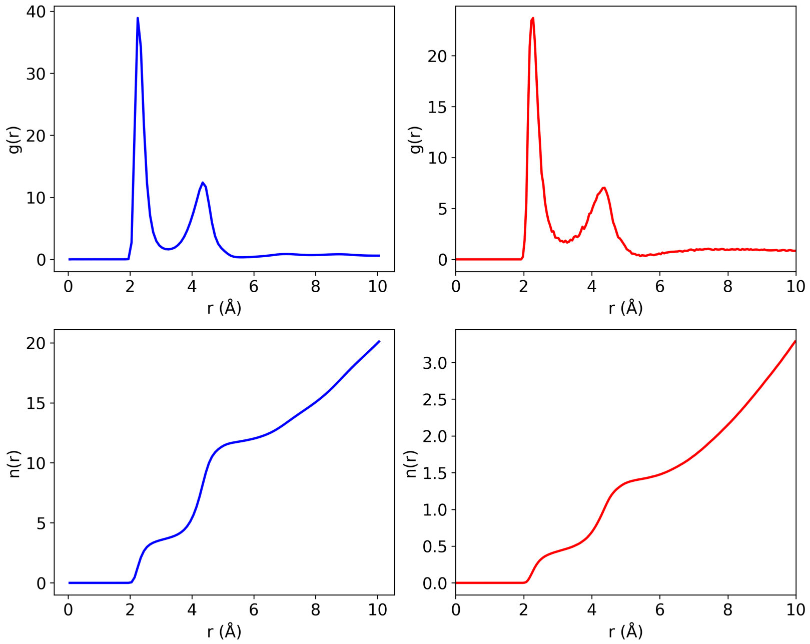

Figure S5: Radial distribution functions, $g(r)$ (upper panels), and integrated coordination numbers, $n(r)$ (lower panels), for PF_6 molecules in the bulk phase (blue line) and at the interface (red line).

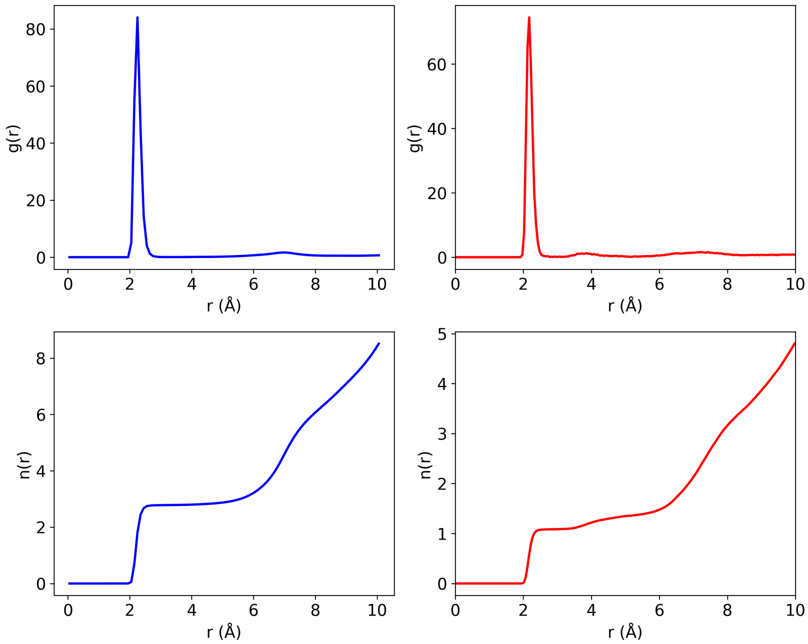

Figure S6: Radial distribution functions, $g(r)$ (upper panels), and integrated coordination numbers, $n(r)$ (lower panels), for TEP molecules in the bulk phase (blue line) and at the interface (red line).

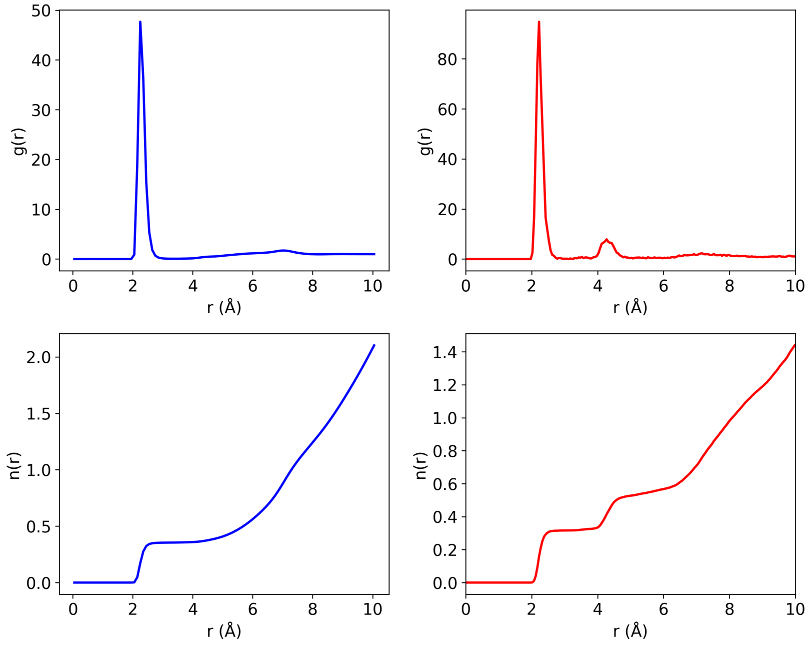

Figure S7: Radial distribution functions, $g(r)$ (upper panels), and integrated coordination numbers, $n(r)$ (lower panels), for DMC molecules in the bulk phase (blue line) and at the interface (red line).

2.3 Exploring Reaction Pathways in the Nanoreactor

Reduction reaction pathways were explored using the nanoreactor approach implemented in xTB, in which molecules are confined within a spherical log-Fermi potential to promote reactive events increasing the number of collisions between species and sodium atom. This strategy accelerates rare bond-breaking processes while preventing unphysical dissociation. For statistical reliability, multiple independent replicas were performed: 20 for TEP, 10 for $PhCF_3$, and 3 for DMC. Reaction events were monitored through time-resolved bond order analysis and fragment charge evolution.

For TEP and $PhCF_3$, MD simulations were carried out at 400 K for 10 ps with a 1.0 fs timestep. The spherical confinement was automatically defined to enclose all atoms. No SHAKE constraints were applied.

For DMC, enhanced sampling was required due to its higher energetic stability. MD were performed at 800 K for 10 ps with a reduced timestep of 0.5 fs and increased hydrogen mass (hmass = 3) to ensure numerical stability. In addition, metadynamics with smooth Gaussian bias potentials (keywords: kpush = 0.01, alp = 2.0) was applied to C-O bonds to facilitate exploration of carbonate dissociation.

2.3.1 TEP + Na reaction

Across the 20 replicas at 400 K, the dominant reactive pathway corresponded to ethyl–phosphate (C–O) bond cleavage. Bond cleavage was identified by a decrease in bond order followed by fragments separation. Charge analysis revealed that the system dissociates into a Na^+ cation, a phosphate anion, and an ethyl radical.

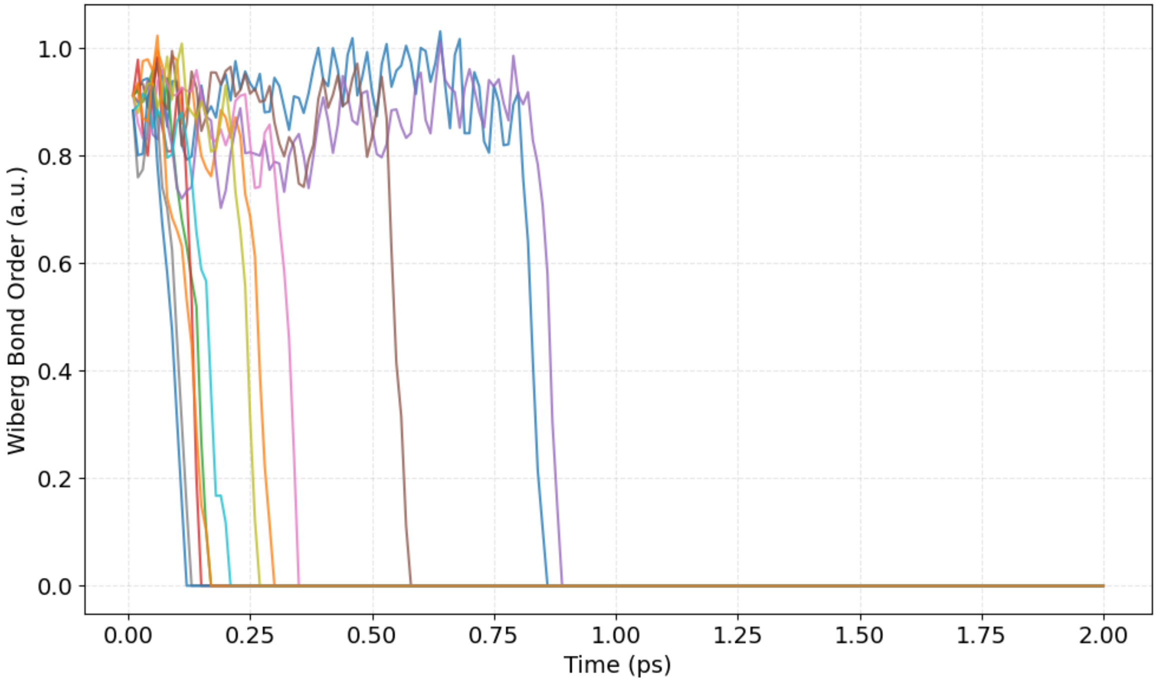

Figure S8: Time evolution of the C–O Wiberg bond order for all replicas of the TEP + Na reaction, showing that bond cleavage occurs very rapidly (< 1.0 ps).

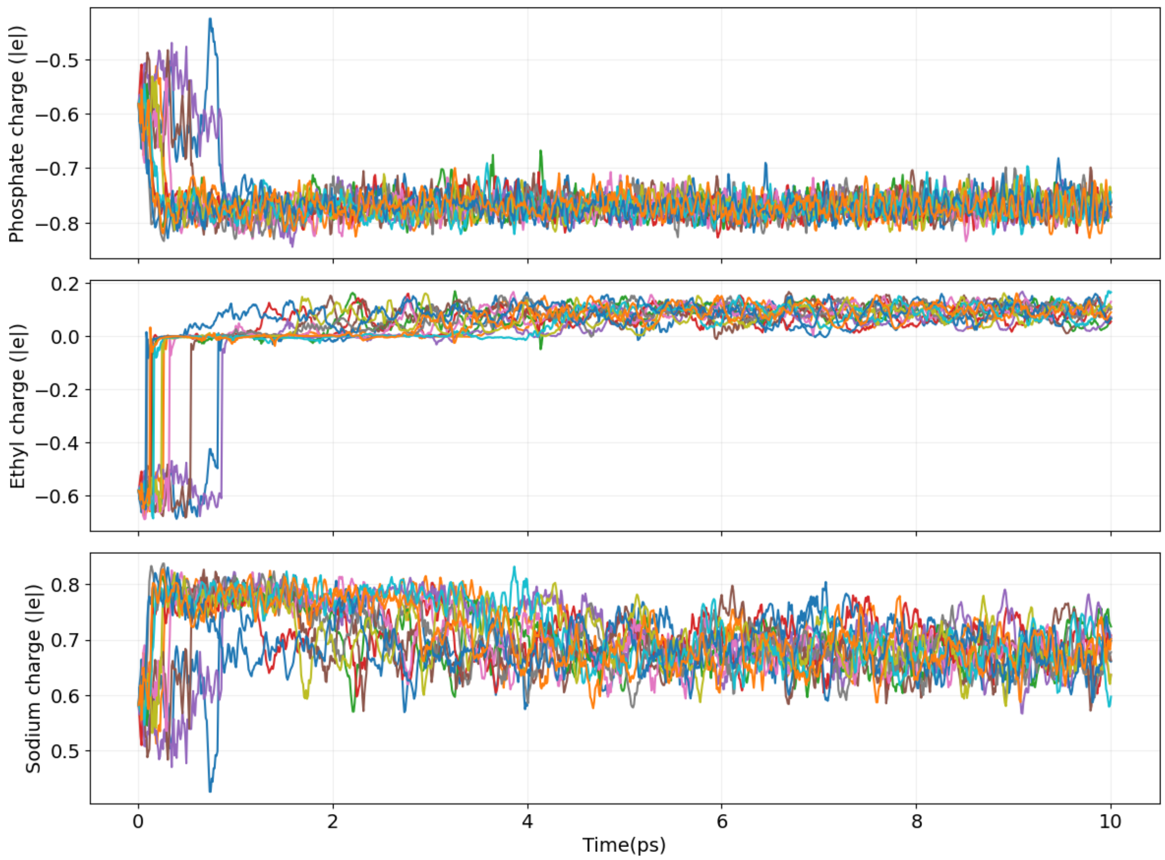

Figure S9: Time evolution of fragment charges for all replicas of the TEP + Na reaction. After 6 ps, the charge values clearly indicate the formation of a phosphate anion (top panel), a neutral ethyl radical (middle panel), and a sodium cation (bottom panel).

2.3.2 $PhCF_3$ + Na reaction

For $PhCF_3$, the event observed among the 10 replicas was C–F bond cleavage, generating a $PhCF_2$ radical and fluoride anion. The aromatic ring remained structurally stable, while heterolytic C–F dissociation was evidenced by bond order decay and charge accumulation on fluorine atom.

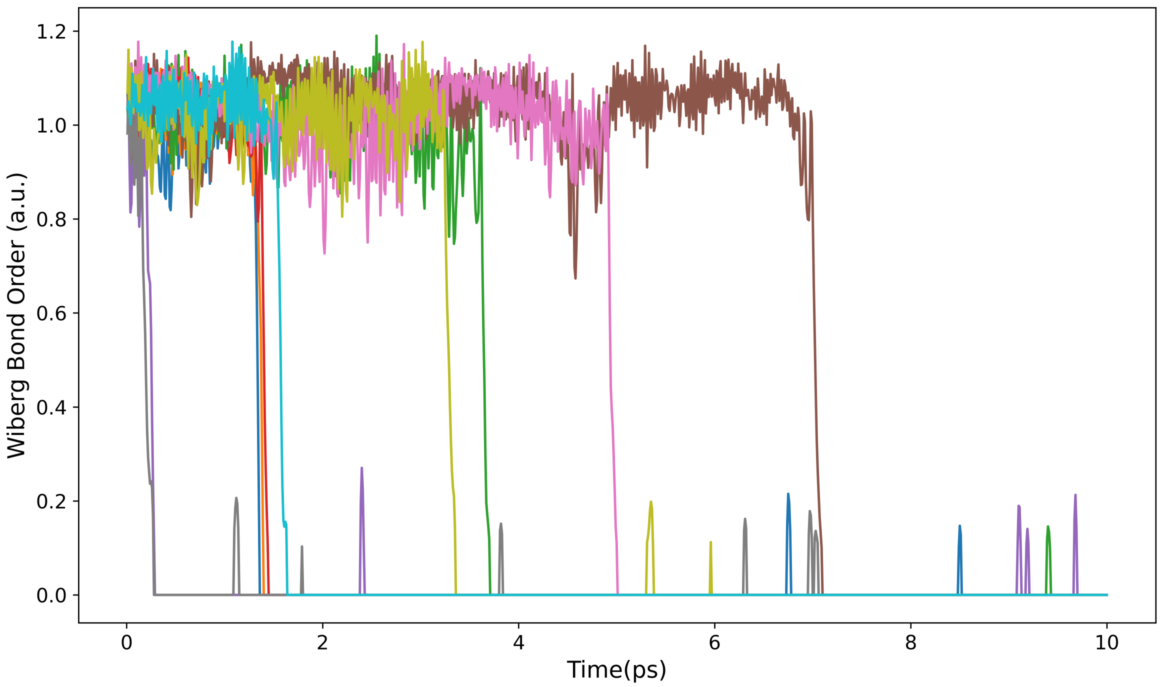

Figure S10: Time evolution of the C–F Wiberg bond order for all replicas of the $PhCF_3$ + Na reaction, showing that all bond cleavage events occur within 8.0 ps of the nanoreactor MD simulations.

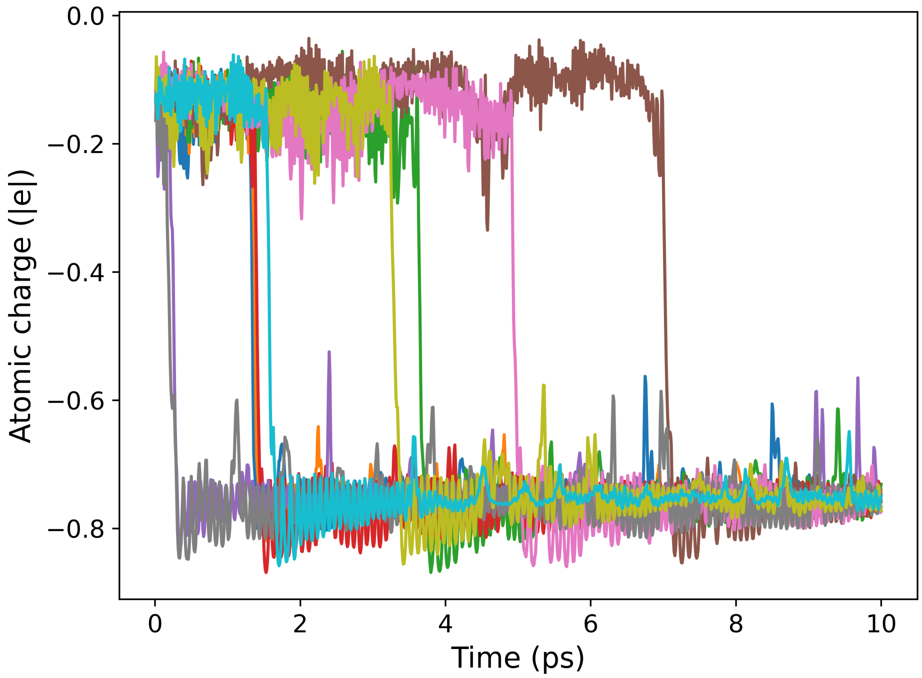

Figure S11: Time evolution of the fluorine atom charge for all replicas of the $PhCF_3$ + Na reaction, indicating the formation of a fluoride anion after 8 ps.

2.3.3 DMC + Na reaction

At 800 K under metadynamics bias, DMC predominantly underwent carbonate–CH_3 (O–CH_3) bond cleavage.

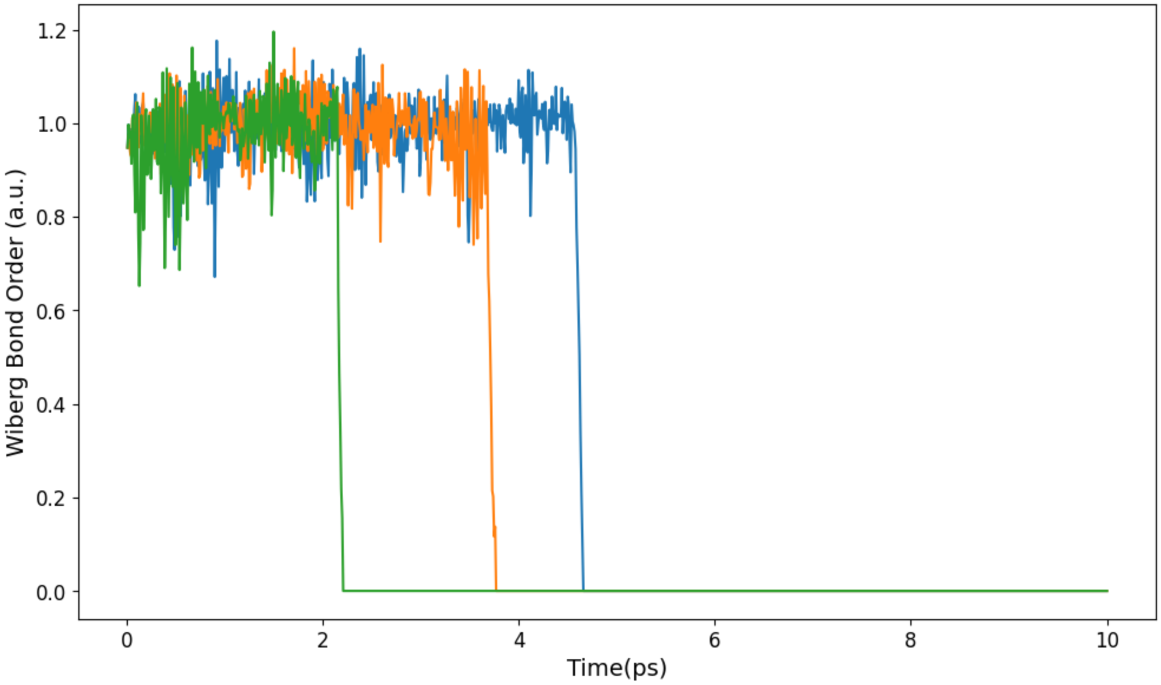

Figure S12: Time evolution of the O–CH_3 Wiberg bond order for all replicas of the DMC + Na reaction, showing that all bond cleavage events occur within 6.0 ps of the nanoreactor MD simulations.

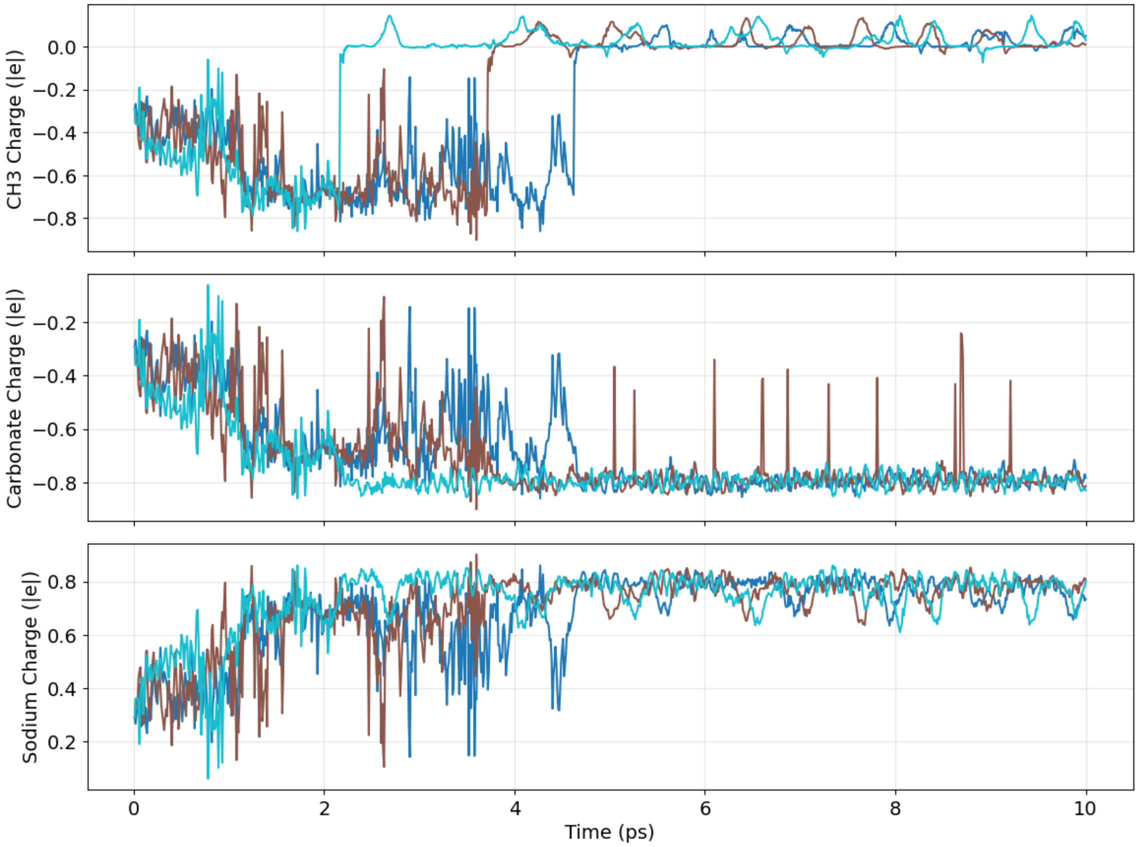

Figure S13: Time evolution of the CH_3 fragment charge for all replicas of the DMC + Na reaction, indicating the formation of a CH_3 fragment after 6 ps.

2.4 Transition-state Calculations

2.4.1 Nudged Elastic Band

The nudged elastic band (NEB) with transition-state optimization (opt-TS) was used to locate the transition state along the reaction pathway, starting from the optimized geometries of the reactant and product. A NEB calculation was then carried out to generate a chain of interpolated images between these structures, identifying the climbing image near the energy maximum (saddle point). This image was subsequently refined using the opt-TS, and frequency analysis confirmed the presence of a single imaginary frequency, validating the transition-state geometry. All NEB and opt-TS calculations were performed at B3LYP-D4/def2-SVP.

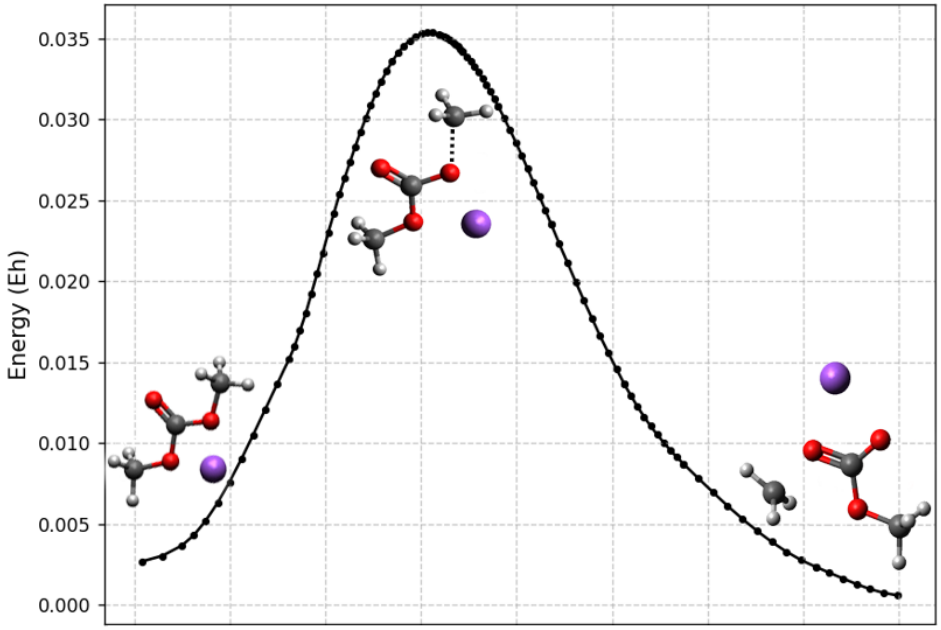

Figure S14: Potential energy profile along the NEB coordinate for the DMC + Na reduction reaction.

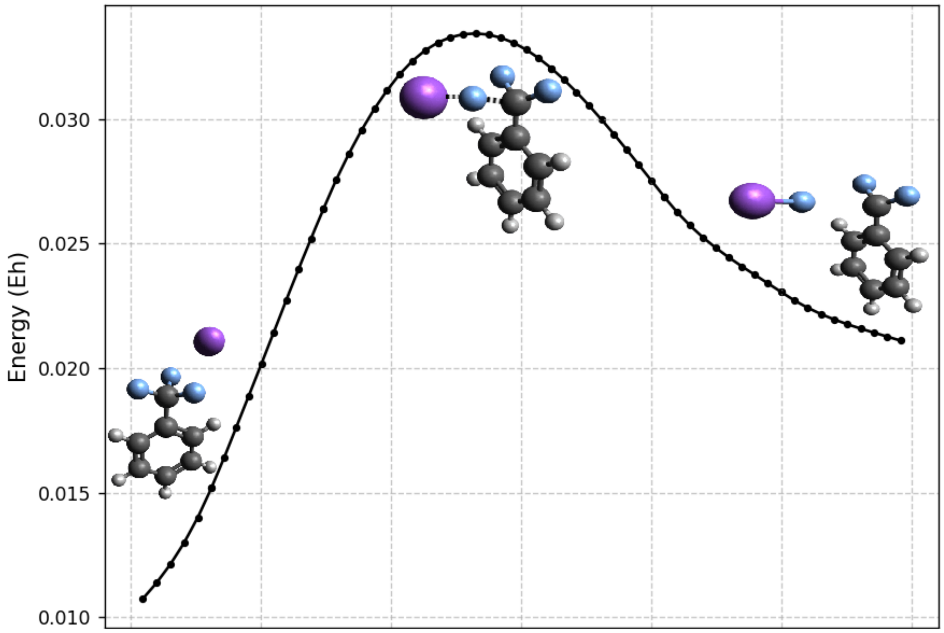

Figure S15: Potential energy profile along the NEB coordinate for the $PhCF_3$ + Na reduction reaction.

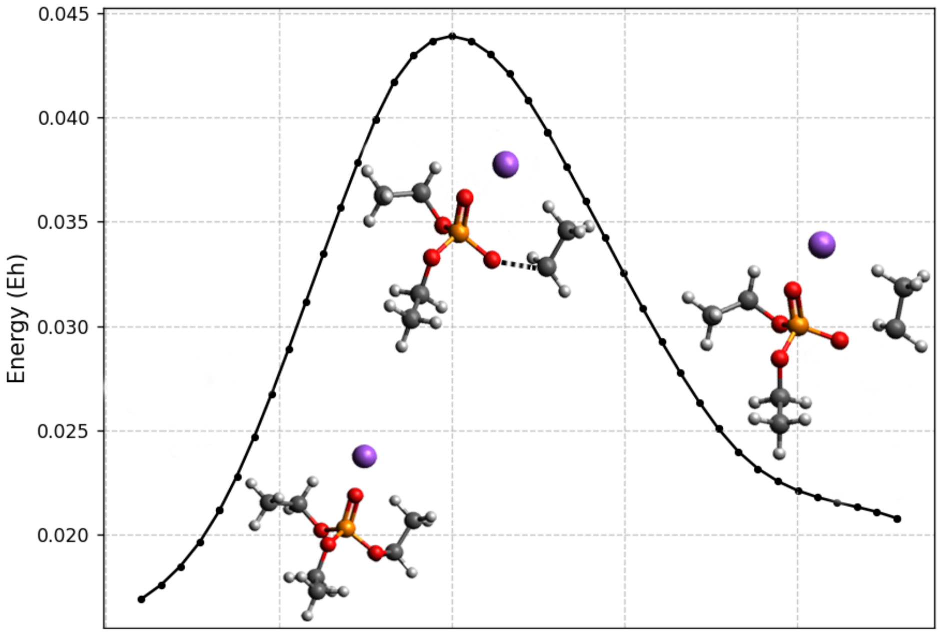

Figure S16: Potential energy profile along the NEB coordinate for the TEP + Na reduction reaction.

2.4.2 Intrinsic Reaction Coordinate

The intrinsic reaction coordinate (IRC) calculations were performed to confirm the connectivity between the optimized transition state and the corresponding reactant and product minima. Starting from the transition-state geometry obtained at opt-TS, the IRC pathway was traced in both forward and backward directions along the mass-weighted reaction coordinate. All IRC calculations were carried out at the B3LYP-D4/def2-SVP level. In addition, single-point calculations in continuum solvent model were performed using C-PCM ($\epsilon_r = 10.0$) with default parameters as implemented in ORCA 5.0.4.

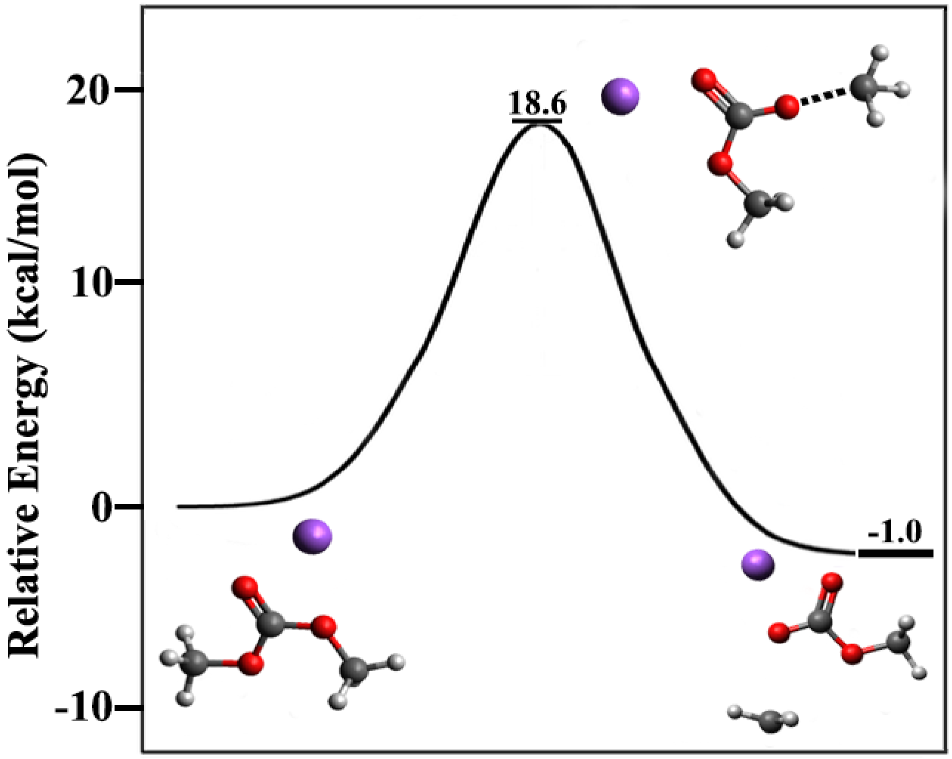

Figure S17: Intrinsic reaction coordinate for the DMC + Na reaction, with the relative energies of the transition state and the product highlighted.

Table S25: Relative energies (in kcal/mol) of the transition-state and product with respect to the reactant state, computed in vacuum and in solvent for the DMC + Na reaction. The reactant, transition state, and product structures were extracted from the IRC pathway (IRC column). Single-point C-PCM calculations were then performed on these IRC structures (IRC-CPCM column).

	IRC	IRC-CPCM
Reagent	0	0
TS	18.6	22.5
Product	-1.1	-6.1

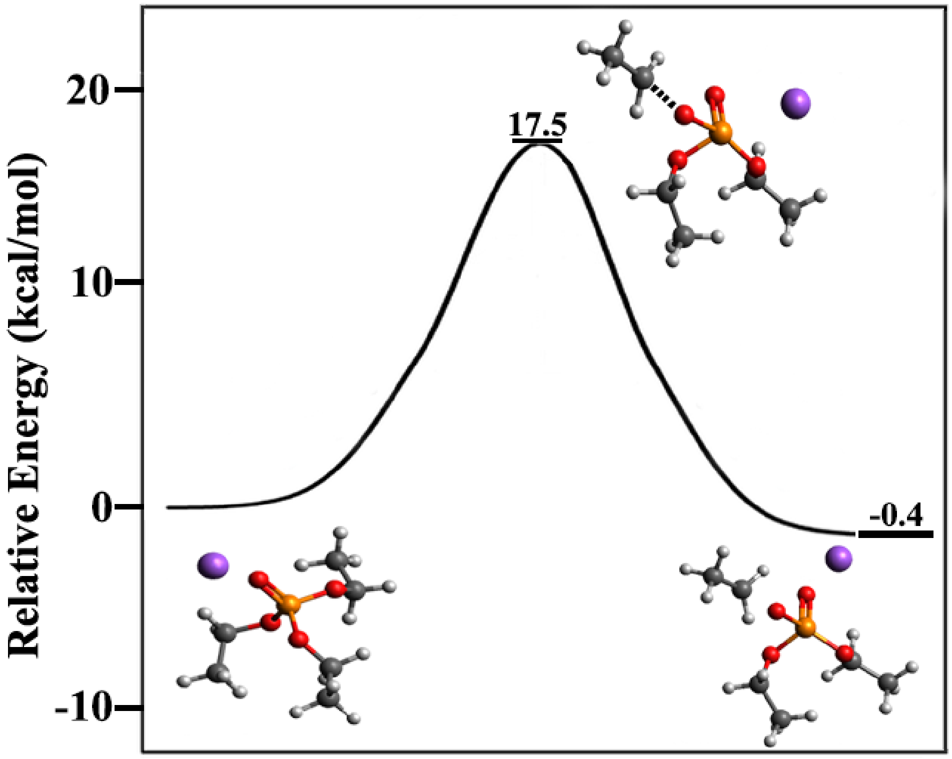

Figure S18: Intrinsic reaction coordinate for the TEP + Na reaction, with the relative energies of the transition state and the product highlighted.

Table S26: Relative energies (in kcal/mol) of the transition-state and product with respect to the reactant state, computed in vacuum and in solvent for the TEP + Na reaction. The reactant, transition state, and product structures were extracted from the IRC pathway (IRC column). Single-point C-PCM calculations were then performed on these IRC structures (IRC-CPCM column).

	IRC	IRC-CPCM
Reagent	0	0
TS	17.5	20.6
Product	-0.4	-7.0

References

[1] K. Vanommeslaeghe, E. Hatcher, C. Acharya, S. Kundu, S. Zhong, J. Shim, E. Darian, O. Guvench, P. Lopes, I. Vorobyov, A. D. Mackerell, Charmm general force field: A force field for drug-like molecules compatible with the charmm all-atom additive biological force fields, Journal of Computational Chemistry 31 (4) (2009) 671–690. doi:10.1002/jcc.21367.
URL http://dx.doi.org/10.1002/jcc.21367

[2] R. M. de Souza, L. J. A. de Siqueira, M. Karttunen, L. G. Dias, Molecular dynamics simulations of polymer–ionic liquid (1-ethyl-3-methylimidazolium tetracyanoborate) ternary electrolyte for sodium and potassium ion batteries, Journal of Chemical Information and Modeling 60 (2) (2019) 485–499. doi:10.1021/acs.jcim.9b00750.
URL http://dx.doi.org/10.1021/acs.jcim.9b00750

[3] J. Fiates, R. H. Ratochinski, T. C. Lourenço, J. L. Da Silva, L. G. Dias, Fluoroalkoxyaluminate-based ionic liquids as electrolytes for sodium-ion batteries, Journal of Molecular Liquids 369 (2023) 120919. doi:10.1016/j.molliq.2022.120919.
URL http://dx.doi.org/10.1016/j.molliq.2022.120919

[4] S. Felletti, M. Spedicato, D. Bozza, C. De Luca, F. Presini, P. P. Giovannini, M. Carraro, M. Macis, A. Cavazzini, M. Catani, A. Ricci, W. Cabri, Dimethyl carbonate as a green alternative to acetonitrile in reversed-phase liquid chromatography. part i: Separation of small molecules, Journal of Chromatography A 1712 (2023) 464477. doi:10.1016/j.chroma.2023.464477.
URL http://dx.doi.org/10.1016/j.chroma.2023.464477

[5] K. Nandhini, N. Cele, B. G. de la Torre, F. Albericio, Triethyl phosphate (tep) as a green solvent for solid-phase peptide synthesis (spps), Green Chemistry Letters and Reviews 17 (1) (Mar. 2024). doi:10.1080/17518253.2024.2330639.
URL http://dx.doi.org/10.1080/17518253.2024.2330639

[6] L. De Lorenzi, M. Fermeglia, G. Torriano, Density and Viscosity of 1-Methoxy-2-Propanol, 2-Methyltetrahydrofuran, alpha,alpha,alpha-Trifluorotoluene, and Their Binary Mixtures with 1,1,1-Trichloroethane at Different Temperatures, Journal of Chemical Engineering Data 41 (5) (1996) 1121–1125. doi:10.1021/je9601220.
URL http://dx.doi.org/10.1021/JE9601220

[7] A. W. Black, P. N. Bartlett, Selection and characterisation of weakly coordinating solvents for semiconductor electrodeposition, Physical Chemistry Chemical Physics 24 (14) (2022) 8093–8103. doi:10.1039/d2cp00696k.
URL http://dx.doi.org/10.1039/d2cp00696k

[8] L. Martínez, R. Andrade, E. G. Birgin, J. M. Martínez, PACKMOL: A package for building initial configurations for molecular dynamics simulations, Journal of Computational Chemistry 30 (13) (2009) 2157–2164. doi:10.1002/jcc.21224.
URL http://dx.doi.org/10.1002/jcc.21224

[9] J. C. Phillips, D. J. Hardy, J. D. C. Maia, J. E. Stone, J. V. Ribeiro, R. C. Bernardi, R. Buch, G. Fiorin, J. Hénin, W. Jiang, R. McGreevy, M. C. R. Melo, B. K. Radak, R. D. Skeel, A. Singharoy, Y. Wang, B. Roux, A. Aksimentiev, Z. Luthey-Schulten, L. V. Kalé, K. Schulten, C. Chipot, E. Tajkhorshid, Scalable molecular dynamics on cpu and gpu architectures with namd, The Journal of Chemical Physics 153 (4) (Jul. 2020). doi:10.1063/5.0014475.
URL http://dx.doi.org/10.1063/5.0014475

[10] T. Darden, D. York, L. Pedersen, Particle Mesh Ewald: An Nlog(N) method for Ewald sums in large systems, The Journal of Chemical Physics 98 (12) (1993) 10089–10092. doi:10.1063/1.464397.
URL http://dx.doi.org/10.1063/1.464397

[11] R. Elber, A. P. Ruymgaart, B. Hess, SHAKE parallelization, The European Physical Journal Special Topics 200 (1) (2011) 211–223. doi:10.1140/epjst/e2011-01525-

9.
URL http://dx.doi.org/10.1140/epjst/e2011-01525-9

[12] A. Brunger, C. L. Brooks, M. Karplus, Stochastic boundary conditions for molecular dynamics simulations of ST2 water, Chemical Physics Letters 105 (5) (1984) 495–500. doi:10.1016/0009-2614(84)80098-6.
URL http://dx.doi.org/10.1016/0009-2614(84)80098-6

[13] S. E. Feller, Y. Zhang, R. W. Pastor, B. R. Brooks, Constant pressure molecular dynamics simulation: The Langevin piston method, The Journal of Chemical Physics 103 (11) (1995) 4613–4621. doi:10.1063/1.470648.
URL http://dx.doi.org/10.1063/1.470648

[14] R. Hafner, G. Guevara-Carrion, J. Vrabec, P. Klein, Sampling the bulk viscosity of water with molecular dynamics simulation in the canonical ensemble, The Journal of Physical Chemistry B 126 (48) (2022) 10172–10184. doi:10.1021/acs.jpcb.2c06035.
URL http://dx.doi.org/10.1021/acs.jpcb.2c06035

[15] Y. Zhang, A. Otani, E. J. Maginn, Reliable Viscosity Calculation from Equilibrium Molecular Dynamics Simulations: A Time Decomposition Method, Journal of Chemical Theory and Computation 11 (8) (2015) 3537–3546. doi:10.1021/acs.jctc.5b00351.
URL http://dx.doi.org/10.1021/acs.jctc.5b00351
